# The efficacy of longevity interventions in *Caenorhabditis elegans* is determined by the early life activity of RNA splicing factors

**DOI:** 10.1371/journal.pbio.3003504

**Published:** 2025-11-21

**Authors:** Sneha Dutta, Maria Camila Perez Matos, Caroline Heintz, Ayse Sena Mutlu, Mary Piper, Meeta Mistry, Arpit Sharma, Christopher S. Morrow, Hannah Smith, Porsha Howell, Rohan Sehgal, Anne Lanjuin, Meng C. Wang, William B. Mair

**Affiliations:** 1 Department of Molecular Metabolism, Harvard T. H. Chan School of Public Health, Harvard University, Boston, Massachusetts, United States of America; 2 Huffington Center on Aging, Baylor College of Medicine, Houston, Texas, United States of America; 3 Howard Hughes Medical Institute, Baylor College of Medicine, Houston, Texas, United States of America; 4 Harvard Chan Bioinformatics Core, Harvard T. H. Chan School of Public Health, Boston, Massachusetts, United States of America; 5 Janelia Research Campus, Howard Hughes Medical Institute, Ashburn, Virginia, United States of America; The Francis Crick Institute, UNITED KINGDOM OF GREAT BRITAIN AND NORTHERN IRELAND

## Abstract

Geroscience aims to target the aging process to extend healthspan. However, even isogenic individuals show heterogeneity in natural aging rate and responsiveness to pro-longevity interventions, limiting translational potential. Using RNAseq analysis of young, isogenic, subpopulations of *Caenorhabditis elegans* selected solely on the basis of the splicing pattern of an in vivo minigene reporter that is predictive of future life expectancy, we find a strong correlation in young animals between predicted life span and alternative splicing of mRNAs related to lipid metabolism. The activity of two RNA splicing factors, Reversed Polarity-1 (REPO-1) and Splicing Factor 1 (SFA-1), early in life is necessary for *C. elegans* response to specific longevity interventions and leads to context-specific changes to fat content that is mirrored by knockdown of their direct target POD-2/ACC1. Moreover, POD-2/ACC1 is required for the same longevity interventions as REPO-1/SFA-1. In addition, early inhibition of REPO-1 renders animals refractory to late onset suppression of the TORC1 pathway. Together, we propose that splicing factor activity establishes a cellular landscape early in life that enables responsiveness to specific longevity interventions and may explain variance in efficacy between individuals.

## Author summary

Efficacy of pro-longevity interventions in *Caenorhabditis elegans* is determined by the activity of splicing factors early in life.

## Abstract 3

Interventions that promote healthy aging differ in their effectiveness between individuals. This study in C. elegans shows that the effect of a pro-longevity intervention can be strongly influenced by the activity of a splicing factor and the lipid metabolic landscape early in an individual's life.

## Introduction

Aging is the key risk factor for most noncommunicable chronic diseases. In the last 20 years, the field of geroscience has discovered genetic, metabolic, and pharmacological pro-longevity interventions that delay aging in multiple species and might be used to promote healthspan in the elderly. These interventions include dietary restriction (DR), intermittent fasting, and genetic or pharmacological modulation of key metabolic and stress response pathways [1]. However, the effectiveness of these interventions is highly variable between individuals, sexes, and genotypes [2]. Given the greater heterogeneity in humans compared to laboratory model organisms, such variance currently limits effective translation of geroscience discoveries to usable therapeutics for the elderly. Understanding the biological mechanisms that underpin heterogeneity of treatment response is therefore needed if we are to leverage the discovery of anti-aging regimens to alleviate diseases of aging in people.

## Results

**Fig 1:**
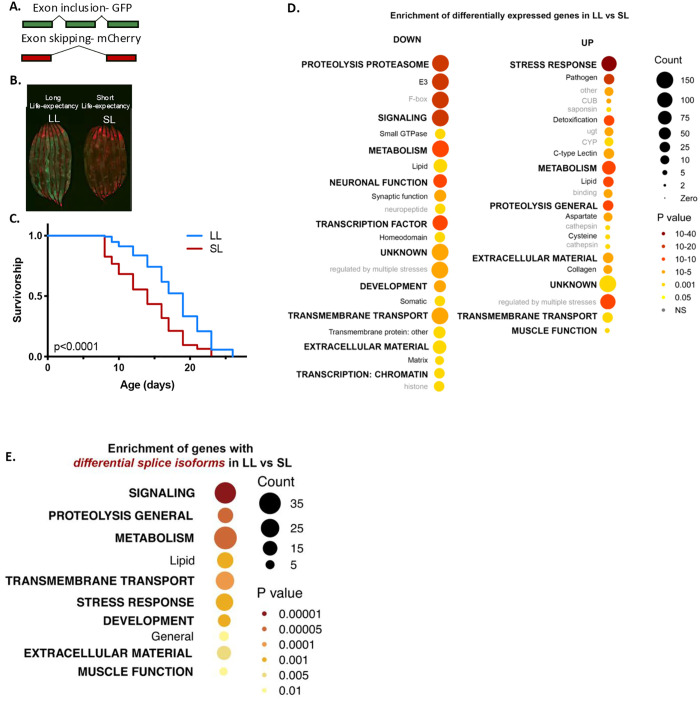
Early life alternative splicing of mRNAs related to lipid metabolism and known longevity pathway correlate with subsequent life expectancy. **A.** Schematic illustrating the fluorescence expression pattern of *ret-1* splicing reporter worm. Inclusion/exclusion of exon 5 results in GFP/mCherry expression, respectively. **B.** Representative images of *ad-libitum* fed *ret-1* reporter worms segregated at Day 6 based on their splicing pattern. **C.** Survivorship of the worm sub-populations separated at Day 6 (*P* < 0.0001, 1 of 6 replicates). Worms that age slowly are marked “LL” (Long Life-expectancy) and worms that age rapidly as “SL” (Short Life-expectancy). **D.** WormCat visualization of categories enriched in down and upregulated genes in LL vs. SL worms. Padj < 0.01 and fold change >1.5 was used as cutoff to mark differentially expressed genes. **E.** WormCat visualization of categories enriched in genes that exhibit differential isoform usage in LL vs. SL worms. Data underlying the graphs in this figure can be found in S1 Data.

Deregulation of pre-mRNA splicing has been implicated in multiple age-related chronic diseases [3], and changes in expression of key splicing regulators and splicing of their targets are associated with longevity in mice and humans [4,5]. Moreover, specific RNA splicing factors have been shown to be causally linked to the effects of pro-longevity interventions in *Caenorhabditis elegans* [6–8]. We therefore hypothesized that changes in alternative splicing of specific pre-mRNAs might be coupled to both the rate of biological aging and response to longevity interventions. To test this, we first sought to define early RNA processing events that correlate with subsequent life expectancy. Fluorescent mini gene reporters of a single exon 5 skipping event in *ret-1* [9] in *C. elegans* can be used to sort isogenic animals at day 6 of adulthood into subpopulations with short or long life (LL) expectancies, identifying animals that are naturally aging poorly or well [6]. Animals with prevalent *ret-1* exon 5 inclusion (GFP) at day 6 show life span extension compared to isogenic animals with exon 5 skipping (mCherry), defined therefore as having LL expectancy versus short life (SL) expectancy, respectively (Fig 1A–1C). We isolated LL or SL subpopulations at day 6 day in sextuplicate (Figs 1C and S1A) and performed 75-bp paired-end RNA-Seq followed by analysis of gene expression and differential isoform usage (S1–S3 Tables). This allowed us to define genes in which either expression level or isoform usage differed between LL versus SL animals.

First, we used WormCat [10] to identify functional signatures in genes significantly up- or downregulated in the LL expectancy animals (Fig 1D; S3 Table). LL animals have decreased expression of multiple functional categories including genes regulating signaling and the proteosome, while genes required for stress response/detoxification and proteolysis, processes known to be longevity-related, are increased. LL animals also show widespread changes in genes required for lipid metabolism, with subsets of lipid metabolic genes showing increased expression, suggesting differences in their lipid metabolism or composition (S1B–S1E Fig). Differential isoform usage between LL and SL animals is enriched for pathways implicated in aging such as signaling, proteolysis, and lipid metabolism further suggesting that splicing of pre-mRNAs specifically linked to these pathways is coupled to life expectancy (Figs 1E and S1F; S4 Table). Notably, differential transcript usage in SL versus LL groups includes genes specific to lipid metabolism, such as *cpt-5*, *cpt-6*, *acox-3*, and *lpb-1*. Instead of a generalized loss of splicing fidelity in animals that are aging poorly, these data suggest that heterogeneity in expression and splicing of genes in specific functional categories, including lipid metabolism and proteolysis, early in the life of an individual is coupled to life expectancy.

If heterogeneity in RNA splicing of specific functional classes of pre-mRNAs is linked to life expectancy, we reasoned that specific RNA splicing-mediated processes may also underlie variation in response to longevity interventions. Previously, we identified causal roles for the RNA splicing factors SFA-1 and REPO-1 in life span extension by DR and suppression of the target of rapamycin complex 1 (TORC1) in *C. elegans* [6]. REPO-1 is a component of the SF3A subunit in the U2snRNP of the spliceosome whereas SFA-1/SFA-1 is a branch-point binding protein that interacts with the UAF proteins and recruits U2 snRNP in the 3′ splice site recognition during early spliceosome assembly [11]. The link between DR and RNA splicing appears conserved, as upregulation of spliceosome components including SF3A in the liver is a signature of DR in rhesus monkeys [12]. We sought to define how these RNA splicing factors causally modulate aging, and whether splicing status in an individual might impact its responsiveness to various pro-longevity interventions.

**Fig 2:**
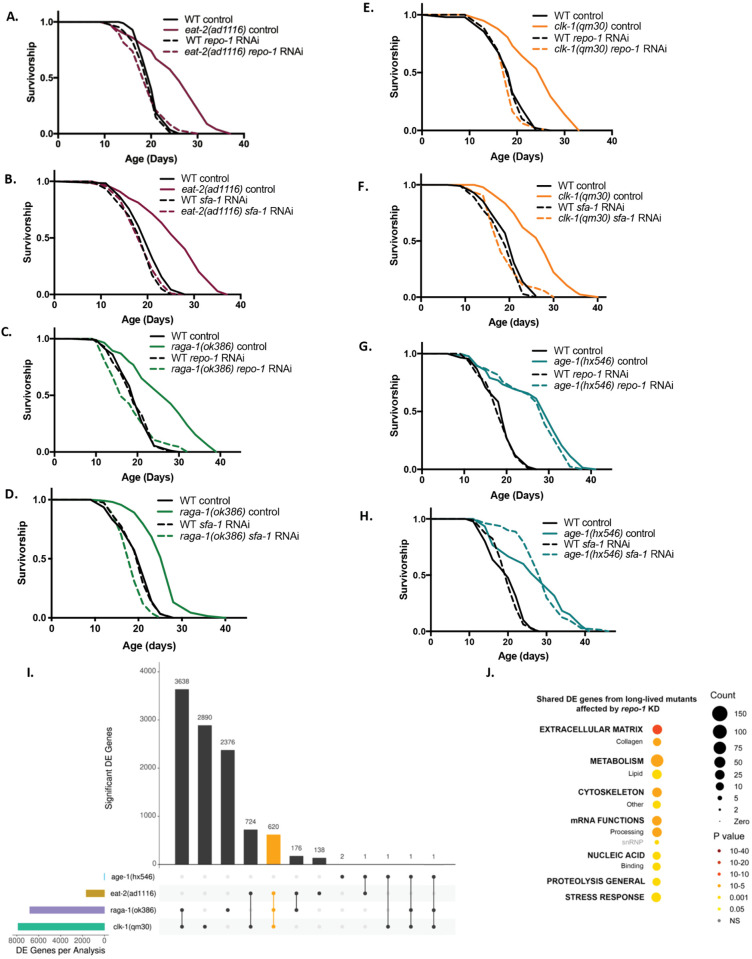
REPO-1 and SFA-1 are required for life span extension in DR, TORC1, and ETC mutant longevity but dispensable for IIS longevity. **A, B.** Survivorship of wild-type (WT) and *eat-2(ad1116)* worms −/+ *repo-1* RNAi (*P* = 0.8753) and *sfa-1* RNAi (*P* = 0.5658) (*p-*values comparing wild-type N2 on RNAi vs. *eat-2(ad1116)* on RNAi, ≥ 3 replicates). **C, D.** Survivorship of WT and *raga-1(ok386)* worms −/+ *repo-1* RNAi (*P* = 0.5407) and *sfa-1* RNAi (*P* = 0.002) (*p-*values comparing wild-type N2 on RNAi vs. *raga-1(ok386)* on RNAi, ≥3 replicates). *repo-1* and *sfa-1* RNAi were administered from hatch. **E, F.** Survivorship of wild-type (WT) and *clk-1(qm30)* worms −/+ *repo-1* RNAi (*P* = 0.1838 and *sfa-1* RNAi (*P* = 0.86) (*p-*values comparing wild-type N2 on RNAi vs. *clk-1(qm30)* on RNAi, ≥3 replicates). *repo-1* and *sfa-1* RNAi were administered from hatch. **G, H.** Survivorship curve of wild-type (WT) and *age-1(hx546)* worms −/+ *repo-1* RNAi (*P* < 0.0001) and *sfa-1* RNAi (*P* < 0.0001) (*p-*values comparing wild-type N2 on RNAi vs. *age-1(hx546)* on RNAi, ≥3 replicates). *repo-1* and *sfa-1* RNAi were administered from hatch. I. UpSet plot (UpSetR R package) quantifying genes in different longevity mutants that respond differently on *repo-1* knockdown compared to wild-type N2 worms. Category of genes shared by SF-dependent mutants are highlighted in yellow. J. WormCat visualization of categories enriched in 620 shared genes that are differentially affected on loss of REPO-1 in splicing factor-dependent pathways compared to wild-type N2 worms. Data underlying the graphs in this figure can be found in S2 Data.

To define whether REPO-1 and SFA-1 activity modulates the efficacy of all longevity interventions or shows specificity, we designed RNAi clones that inhibit REPO-1 or SFA-1 in *C. elegans* without impacting neighboring gene expression (S2 Fig). Using these specific RNAi clones, we examined the role of REPO-1 and SFA-1 in a series of long-lived *C. elegans* mutants, targeting distinct longevity pathways. RNAi of REPO-1 and SFA-1 fully suppresses life span extension via the DR mimic *eat-2(ad1116)* (Fig 2A and 2B), and mutations to multiple components of the TORC1 pathway (*raga-1(ok386)*, *rsks-1(ok1255)*) (Figs 2C, 2D, and S3A) [6] and electron transport machinery (*clk-1(qm30)*, *isp-1(qm150)*, *nuo-6(qm200)*) (Figs 2E, 2F and S3B–S3D). However, knockdown of REPO-1 or SFA-1 had no effect on life span extension via mutations to multiple components of the insulin/insulin-like growth factor signaling (IIS) pathway (*age-1(hx546)/*PI3K, *daf-2(e1370)/*InR) (Figs 2G, 2H, and S3E) [6], despite blocking life span extension by overexpression of the IIS mediator DAF-16/FOXO (S3F and S3G Fig). We confirmed *repo-1* RNAi inhibited *repo-1* expression equally in both longevity-responsive and nonresponsive mutations (S3H Fig). REPO-1 and SFA-1 activity thus determines responsiveness to specific longevity interventions, rather than modulating global changes that render animals refractory to life span extension by any means.

We leveraged this longevity intervention specificity to define the mechanisms underpinning the effects of REPO-1 on lifespan extension. We examined the transcriptional effects of *repo-1* RNAi on wild-type (WT) animals and a panel of long-lived mutants that either require REPO-1 and SFA-1 for their effects (splicing factor “SF”-dependent) or are refractory to them (SF-independent). We performed four replicates of 75-bp paired-end RNA-Seq on WT (N2), three SF-dependent mutants, *eat-2(ad1116)*, *raga-1(ok386)*, *clk-1(qm30)*, and one SF-independent mutant, *age-1(hx546)*, with and without *repo-1* RNAi (S5 Table). Principal component analysis showed that the transcriptomes of WT, *age-1(hx546)* and *raga-1(ok386)* cluster most tightly, suggesting they share more similarity to each other than to *eat-2(ad1116)* and *clk-1(qm30)* (S3I Fig). Interestingly, *repo-1* RNAi drove significant changes specifically in *eat-2(ad1116)*, *raga-1(ok386),* and *clk-1(qm30)* mutants yet had a less pronounced effect on WT and *age-1(hx546)* worms, closely mirroring the specific longevity effects of *repo-1* knockdown (S3I Fig). To identify those changes in gene expression resulting from loss of *repo-1* that are specific to each long-lived mutant, we compared the transcriptional effects of *repo-1* knockdown on each long-lived mutant to the effects of *repo-1* knockdown on WT worms. We identified genes whose expression responded differently to *repo-1* RNAi in the long-lived mutants compared to WT (S6 Table). These genes were further categorized and grouped based on the mutant in which they were found (Fig 2I). Interestingly, in contrast to the widespread differences induced by *repo-1* RNAi in each of the SF-dependent mutants, only 6 genes responded differently on *repo-1* knockdown in the SF-independent mutant *age-1(hx546)* compared to WT worms (Fig 2Iand S6 Table). Together, these data suggest that REPO-1 has unique functional roles in the physiology of SF-dependent mutants not seen in WT or IIS mutants, further supporting the idea that REPO-1 activity mediates responsiveness to specific longevity interventions.

We sought to identify the unique role of REPO-1 in SF-dependent longevity interventions (Fig 2I yellow). We reasoned that processes by which REPO-1 mediates longevity might be reflected in the 620 genes that are differentially regulated by *repo-1* RNAi across all SF-dependent long-lived mutants (S6 Table). We used the WormCat platform [10] to identify signatures in these shared 620 differentially regulated genes. REPO-1 dependent gene categories enriched across all splicing sensitive pathways included RNA processing, suggesting indirect or compensatory changes in the posttranscriptional machinery on loss of REPO-1 specifically in these longevity pathways. Strikingly, metabolism was one of the most significantly enriched REPO-1 dependent terms, with lipid metabolism being one of the most enriched sub-categories (Figs 2J and S4A–S4D; S7 Table). These data mirror processes coupled to heterogeneity in life expectancy (Fig 1). In addition, we showed previously that loss of SFA-1 specifically reverses expression of lipid metabolism genes induced by DR [6]. Altogether, these data suggest altered lipid metabolism is associated with the effect of REPO-1 and SFA-1 on the aging process and response to treatment.

**Fig 3:**
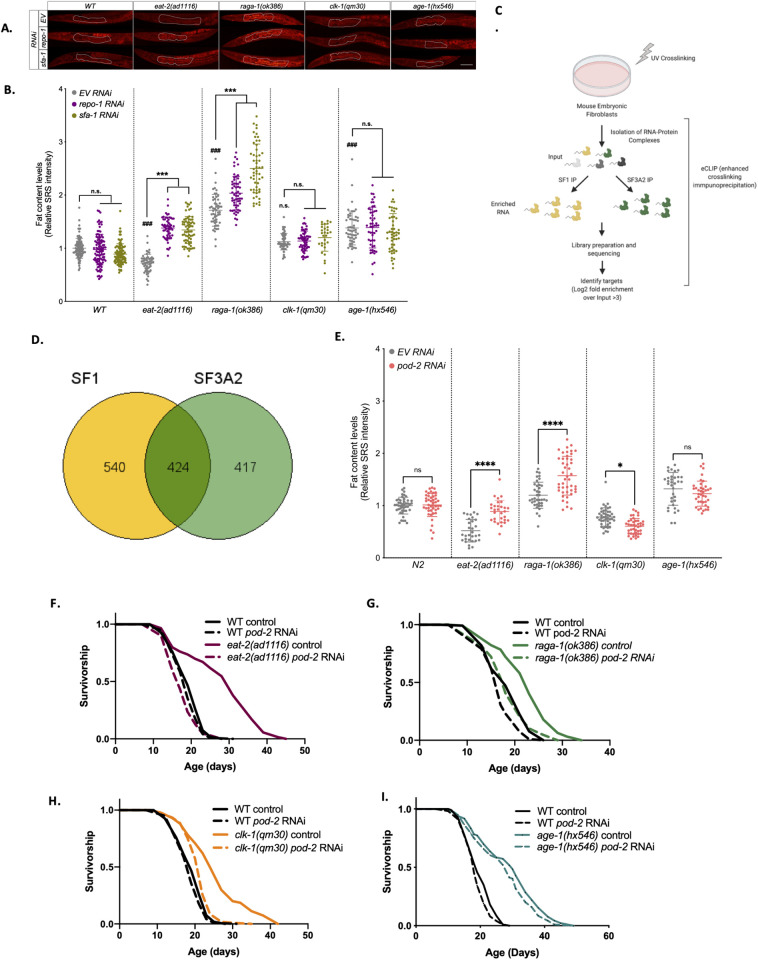
Loss of REPO-1 specifically affects lipid metabolism in splicing factor-sensitive longevity pathways. **A.** Representative images of SRS microscopy showing fat levels in wild-type N2, *eat-2(ad1116)*, *raga-1(ok386)*, *clk-1(qm30)*, and *age-1(hx546)* on control, *repo-1* and *sfa-1* RNAi. Worms were fed on RNAi from hatch and imaged 24 hours post-L4 stage. **B.** Quantification of SRS signal using ImageJ (pooled data quantifying anterior intestine of *n* = 20−30 worms, *N* = 3; ### *P*<0.001 long-lived mutant vs. wildtype on control EV RNAi; *** *P*<0.001 control EV vs. *repo-1/sfa-1* RNAi; n.s. *P* > 0.05). **C.** Schematic of enhanced crosslinking immunoprecipitation (eCLIP) in Mouse Embryonic Fibroblasts (MEFs). *Created in BioRender. SHARMA,* A. *(2025)* *https://BioRender.com/0v2egpn*. **D.** Venn diagram showing targets of SF1 (mammalian SFA-1 ortholog) and SF3A2 (mammalian REPO-1 ortholog) and their overlap in MEFs. True gene targets identified as peaks in IP over input sample that had a log2 fold enrichment > 2 and −log10(*p*-value) > 2, *N* = 2. Plot generated using GeneVenn. **E.** Quantification of SRS signal using Image J. Animals were fed with *pod-2* RNAi at day 1 and imaged at day 4 of adulthood (pooled data quantifying anterior intestine of *n* = 10−30 *Caenorhabditis elegans*, *N* = 3, ***P<0.001 control/empty vector vs. *pod-2* RNAi; *P = 0.0256; n.s *P* > 0.05). **F–I.** Survivorship curves −/+ *pod-2* RNAi of wildtype (WT) and **F.** *eat-2(ad1116)* (*P* = 0.057, 2 replicates); **G.** *raga-1(ok386)* (*P* = 0.1923, 4 replicates); **H.** *clk-1(qm30)* (*P* < 0.0001, 2 replicates), and **I**. *age-1(hx546)* (*P* < 0.0001, 3 replicates). RNAi initiated at Day 1 of adulthood. *p*-values reflect comparison of wild-type N2 fed with *pod-2* RNAi vs. long-lived mutant with *pod-2* RNAi. Data underlying the graphs in this figure can be found in S3 Data.

Next, we examined directly how lipid levels are impacted by *repo-1* and *sfa-1* RNAi in WT and long-lived mutants. Stimulated Raman scattering (SRS) microscopy in live animals showed that neither *sfa-1* and *repo-1* RNAi had any effect on lipid content in the intestine of WT *C.* elegans, yet significantly increased lipid levels in the SF-dependent mutants *eat-2(ad1116)* and *raga-1(ok386)* mutants (Fig 3A and 3B). Lipid levels were not affected by loss of REPO-1 or SFA-1 in the splicing-resistant mutant *age-1(hx546)*. Interestingly, *sfa-1* and *repo-1* RNAi also had no effect on the lipid content of the splicing-sensitive *clk-1(qm30)* mutant, which have impaired mitochondrial function (Fig 3B), suggesting the mechanism by which these splicing factors impact lipid levels might be linked to mitochondrial function. To further characterize the effect of *repo-1* knockdown on lipid metabolism, we performed lipidomics by LC/MS in the long-lived mutants with and without *repo-1* RNAi. (S8 Table). We did not observe major differences in the lipidome between strains or with *repo-1* RNAi (S5A Fig) Interestingly, neither WT nor *age-1(hx546)* had any lipid species that significantly changed upon *repo-1* knockdown, while all SF-dependent lines, *eat-2(ad1116), raga-1(ok386)*, and *clk-1(qm30)* did, suggesting that the *repo-1* effect on lipid metabolism may be specific to SF-dependent pathways in which REPO-1 is required for longevity (S5C Fig). We examined total triacylglycerol concentration and, analogous to our SRS microscopy, *eat-2(ad1116)* and *raga-1(ok386)* trended toward increased triacylglycerides upon loss of REPO-1, although only *raga-1(ok386)* changes were significant (*p*-value 0.02) (S5B Fig). Together, these data suggest that the activity of these splicing factors not only specifically regulates expression and splicing of lipid metabolic genes in SF-dependent interventions but also lipid content itself. However, we did not identify a consensus group of lipid species that changed in all SF-dependent mutants. Thus, we looked for shared regulators of lipid metabolism that might represent a causal link between SFA-1/REPO-1 and longevity.

To understand the mechanism by which SFA-1 and REPO-1 modulate lipid remodeling and longevity, we identified their direct pre-mRNA targets by enhanced cross-linking immunoprecipitation (eCLIP) [13]. We performed eCLIP in both *C. elegans* and mouse embryonic fibroblast (MEF) cell lines, speculating that true targets that impact life span would be shared between the two splicing factors and conserved across organisms. MEFs were cross-linked followed by IP with SF1 (mammalian SFA-1) and SF3A2 (mammalian REPO-1) antibodies (Fig 3C). True peaks were defined as those showing enrichment of log2 fold change ≥ 2 and −log10(*p*-value) ≥ 2 over input (S9 Table). Sequencing identified ~964 and ~841 genes targeted by SF1 and SF3A2 in MEFs, respectively. Four hundred twenty-four genes, representing ~50% of total targets, are shared, suggesting that SF1 and SF3A2 act together to regulate splicing (Fig 3D; S9 Table). eCLIP in *C. elegans* shows that many targets of REPO-1 and SFA-1 are also shared in worms, including *tos-1* (Target of Splicing 1), a known and direct target of SFA-1 [14,15] (S4E–S4G Fig; S10 Table). Interestingly, in both MEFs and in *C. elegans*, we find an enrichment of shared target genes involved in RNA processing and in lipid metabolism. One such target involved in lipid metabolism is Acetyl CoA Carboxylase 1 (ACC1), the rate-limiting enzyme of the fatty acid biosynthetic pathway. ACC1 is a target of both SF1 and SF3A2 in MEFs (S8 Table), and the pre-mRNA for POD-2, the *C. elegans* orthologue of ACC1, was identified as a shared target of SFA-1 and REPO-1 in *C. elegans* (S10 Table).

POD-2/ACC1 converts acetyl-CoA to malonyl-CoA, thus catalyzing the first step in the formation of de novo lipids. We therefore reasoned that dysfunctional POD-2/ACC1 might alter lipid stores in SFA-1 and REPO-1-depleted animals, and that this might modulate the aging effects in SF-sensitive pro-longevity mutants. To test this, first, we measured lipid content changes through SRS microscopy on the different longevity mutants upon *pod-2* RNAi. *pod-2* is the ortholog of human ACACA (acetyl-CoA carboxylase alpha), which catalyzes the rate-limiting step in fatty acid synthesis. We therefore hypothesized that upon POD-2 knockdown, lipid content would be suppressed. However, in contrast, POD-2 knockdown phenocopies REPO-1 knockdown (Fig 3E); with *eat-2*(ad1116) and *raga-1*(ok386) animals showing increased lipid content upon POD-2 knockdown, and no effect in WT or *age-1*(hx546) animals, or in *clk-1(qm30)*, a background that is splicing factor sensitive for its effects on longevity, but shows no changes to lipid content by SRS with knockdown of *sfa-1* and *repo-1*. Next, we inhibited life span extension of SF-dependent and SF-independent mutants with and without RNAi of ACC/POD-2. Again, inhibition of POD-2 mirrors completely the longevity intervention specificity of SFA-1 and REPO-1. *pod-2* RNAi from day 1 of adulthood fully suppresses life span extension of SF-sensitive *eat-2(ad1116)*, *raga-1(ok386)*, and *clk-1(qm30)* (Fig 3F–3H) mutants but does not suppress life span in the SF-resistant *age-1(hx546)* (Fig 3I) mutants. Together, these data reveal a striking similarity in the specificity of both the lipid accumulation phenotypes and the responses to specific longevity interventions resulting from knockdown of SFA-1/REPO-1 and by knockdown of their direct target ACC/POD-2. However, our finding that not all SF-sensitive interventions show parallel changes to lipid content or profile after knockdown of *repo-1* or *sfa-1* (or *pod-2*) suggests that the underlying mechanism by which splicing factor activity confers longevity may be more complex than through gross changes to lipid abundance.

**Fig 4:**
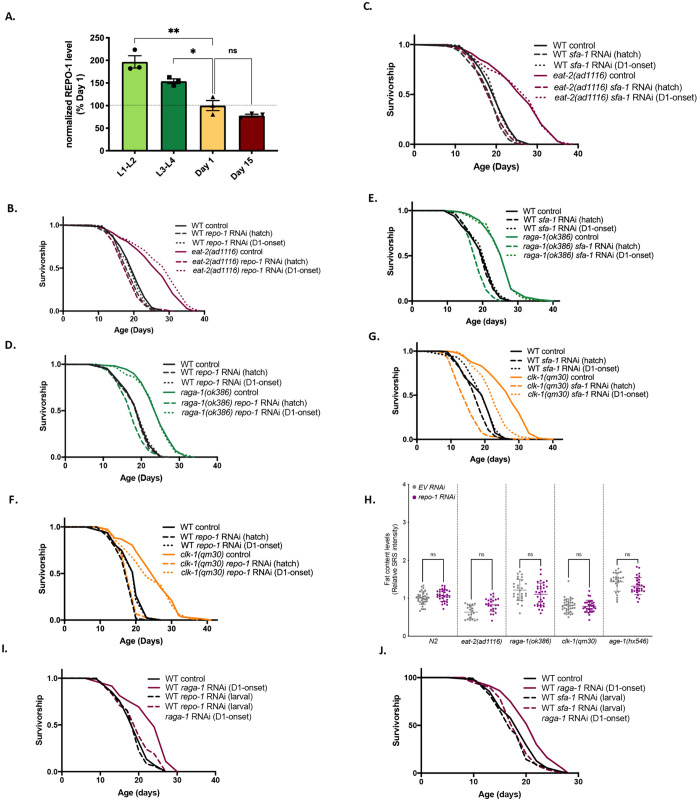
REPO-1 and SFA-1 create a permissive landscape during the early stages of development to mediate longevity benefits later in life. **A.** Quantification of bands from western blot of CRISPR-tagged endogenous 3XFLAG::REPO-1 worms at early (L1-L2) and late (L3-L4) larval stages and at Day 1 and Day 15 of adulthood. Blots probed with 3XFLAG and actin antibodies. Quantification done using ImageJ, normalized to intensity of actin band and plotted as percent of expression at Day 1 of adulthood (**** *P* ≤ 0.0001, *** *P* ≤ 0.001, ** *P* ≤ 0.01, * *P* ≤ 0.05; ns *P* > 0.05; *n* = 3). **B–G.** Survivorship of wild-type (WT) and long-lived mutants **B.** *eat-2(ad1116)*; **D.** *raga-1(ok386)*, and **F.** *clk-1(qm30)* −/+ *repo-1* from hatch (- - -) or Day 1 of adulthood (D1-onset) (…). *P *= 0.5315, 0.0285, 0.2672 for WT *repo-1* RNAi hatch vs. *eat-2(da1116)*, *raga-1(ok386)* and *clk-1(qm30)* mutants on *repo-1* RNAi hatch respectively). *P* = <0.0001, <0.0001, <0.0001 for WT *repo-1* RNAi D1-onset vs. *eat-2(da1116)*, *raga-1(ok386)*, and *clk-1(qm30)* mutants on *repo-1* RNAi D1-onset, respectively). Survivorship of wild-type (WT) and long-lived mutants **C.** *eat-2(ad1116)*; **E.** *raga-1(ok386)*, and **G.** *clk-1(qm30)* −/+ *sfa-1* from hatch (- - -) or Day 1 of adulthood (D1-onset) (…). *P *= 0.5658, 0.002, <0.0001 for WT *sfa-1* RNAi hatch vs. *eat-2(da1116)*, *raga-1(ok386)*, and *clk-1(qm30)* mutants on *sfa-1* RNAi hatch, respectively). *P* = <0.0001, <0.0001, <0.0001 for WT *sfa-1* RNAi D1-onset vs. *eat-2(da1116)*, *raga-1(ok386)*, and *clk-1(qm30)* mutants on *sfa-1* RNAi D1-onset, respectively). **H.** Quantification of SRS signal using ImageJ (pooled data quantifying anterior intestine of *n* = 20−30 worms, *N* = 3; n.s. *P* > 0.05 control EV vs. *repo-1*RNAi). repo-1 RNAi was started on day 1 of adulthood and images were taken on day 4. **I.** Survivorship curve of wild-type N2 worms −/+ *repo-1* RNAi in the larval stages −/+ *raga-1* RNAi from Day 1 of adulthood (*P* = 0.0244 wild-type N2 on *repo-1* RNAi (larval) vs. wild-type N2 on *repo-1* RNAi (larval)+ *raga-1* RNAi (D1-onset), 3 replicates). **J.** Survivorship curve of wild-type N2 worms −/+ *sfa-1* RNAi in the larval stages −/+ *raga-1* RNAi from Day 1 of adulthood (*P* = 0.2480 wild-type N2 on *sfa-1* RNAi (larval) vs. wild-type N2 on *sfa-1* RNAi (larval)+ *raga-1* RNAi (D1-onset), 2 replicates). Data underlying the graphs in this figure can be found in S4 Data.

Disruption of mitochondrial ETC function and DR/TORC1 inhibition operate in temporally distinct time windows to slow aging in *C. elegans*. Knockdown of ETC complexes only during the L3–L4 stages of larval development increases longevity, while DR and TORC1 inhibition confer longevity benefits in adulthood [16,17]. To understand how REPO-1 and SFA-1 modulate longevity and lipid metabolism across seemingly mechanistically unrelated longevity interventions, we asked when in life these splicing factors act to modulate life span. We fluorescently tagged endogenous REPO-1 and SFA-1 by CRISPR knock-in of wrmScarlet and GFP, respectively. Both splicing factors are expressed in nuclei of all cells (S6A Fig), throughout all life stages (S6B Fig), and ages of *C. elegans* (S6C–S6E Fig). Neither *repo-1* mRNA nor REPO-1 total protein diminish with age during adulthood (S6C and S6D Fig). However, REPO-1 levels peak during early development, hinting that splicing factors such as REPO-1 play an important role in early life stages of *C. elegans* (Figs 4A and S6F).

To define the temporal window in which REPO-1 and SFA-1 mediate DR, TORC1, and ETC longevity, we subjected *eat-2(ad1116)*, *raga-1(ok386)*, and *clk-1(qm30)* animals to *repo-1* and *sfa-1* RNAi from hatch or Day 1 of adulthood (D1-onset). Despite western analysis confirming equal knockdown efficiency by day 3 of adulthood in both conditions (S6G–S6J Fig), their effect on longevity was strikingly different; knockdown of both *repo-1* and *sfa-1* in development fully suppresses longevity in all SF-sensitive mutants. However, adult-onset inhibition had no impact on aging regardless of the timing requirement of the SF-dependent intervention itself (Fig 4B–4G). Adult-onset knockdown of REPO-1 also has no effect on the accumulation of lipids as measured by SRS microscopy (Fig 4H). Together, these data suggest REPO-1 and SFA-1 modulate the cellular landscape early in life and that this permissive landscape may contribute to the efficacy of subsequent longevity interventions. Supporting this concept, adult-onset RNAi of *raga-1* is sufficient to prolong life span yet has no effect on animals with prior inhibition of either *repo-1* or *sfa-1* during development (Fig 4I and 4J).

## Discussion

Geroscience approaches over the last 20 years have shown that environmental conditions and genetic manipulation can strongly influence the rate of physiological aging. In particular, DR has a strong beneficial effect in a wide variety of organisms tested to date, not only increasing longevity but also protecting against many chronic diseases. Harnessing the molecular and cellular processes mediating the plasticity of aging in response to DR has the potential to yield novel therapeutics that broadly reduce disease incidence in the elderly. However, while there has been much research into the molecular mechanisms mediating longevity interventions using model organisms, little emphasis has been placed on predicting and understanding variance in their efficacy [2]. This is critical, since the same therapeutic treatment can be beneficial or harmful, depending on the sex, genotype, and physiological state of the individual to which it is applied. For example, dosage levels of pharmacological treatments or DR have very different longevity effects in different genotypes [18,19]. Such genotype by diet interaction may well explain the opposing results of two longitudinal life span studies of DR on rhesus monkeys [20].

Beyond inter-genotype variation to longevity interventions, substantial variation also exists in inter-individual responses. Even within isogenic populations of *C. elegans*, or inbred *Drosophila* and mouse strains, high variance exists both for life span itself and response to DR/DR mimetics. Indeed, for interventions such as methionine restriction, though median life span of mice and rats is increased, a sub-population dies earlier than non-restricted controls [21]. A “one size fits all” approach to DR or DR mimetics is therefore unlikely to be useful therapeutically. Successfully translating geroscience to human application requires accurately predicting a given individual’s response to a given treatment, depending on the genotype and physiology of the individual. Such an approach will allow treatments to be assigned to each individual in a personalized way, maximizing health benefits. The first step toward this end is to identify biological variables that predict individual-specific optimal aging interventions in model organisms, to provide the foundation for a personalized medicine approach to healthy aging therapeutics in humans.

Here, we show that in *C. elegans*, a single RNA exon skipping event can predict the life expectancy of isogenic animals. We leveraged this finding to define functional categories of genes that are differentially expressed or spliced in isogenic animals that long- or short-lived. Animals that naturally age well show gene expression and RNA splicing changes enriched in specific ontology classes, including signaling, proteolysis, and lipid metabolism. We utilized splicing factor-dependent and independent longevity pathways to define RNA splicing-mediated processes coupled to variation in response to anti-aging interventions in *C. elegans*. Regulation of genes tied to lipid metabolism was a shared process correlated to both WT life expectancy and response to treatment. REPO-1 and SFA-1 activity modulate lipid levels in SF-dependent mutants and bind to POD-2/ACC *pod-2* transcripts directly. REPO-1, SFA-1, and POD-2 specifically modulate efficacy of the same pro-longevity mutations and show similar effects on lipid content. Lastly, we show that modulating the activity of REPO-1 and SFA-1 early in life determines whether subsequent application of a pro-longevity intervention, in this case inhibition of RAGA-1, successfully slows aging.

Our data reveal a link between the early life activity of splicing factors and the efficacy of certain longevity interventions, as well as to context-dependent changes in lipid accumulation. Yet, we were not able to identify any common lipid signature or change in lipid abundance that links a specific lipid alteration to longevity. More work is needed to determine if a common change in SF-sensitive mutants can explain the role of *pod-2* in life span. Interestingly, POD-2 has also been linked to longevity seen in germlineless *glp-1* mutants, which also have lipid phenotypes [22]. This will likely require further work to dissect the tissues and cell types in which splicing factors and POD-2 act to modulate aging, and more nuanced examination of lipid composition changes at defined locations in those cell types.

This work demonstrates that the efficacy of a pro-longevity intervention can be strongly influenced by prior events, in this case the activity of a splicing factor earlier in the life of the individual. If this phenomenon is conserved in mammals, it raises important questions about how we design and implement anti-aging therapeutics. Ultimately, it might be possible to predict response to treatment and optimize the intervention via analysis of a subset of RNA splicing events. Such an approach is key if we are to move beyond biomarkers of physiological age towards biomarkers that facilitate precision medicine approaches to interventional geroscience.

## Supporting information

S1 Fig Early life alternative splicing of RNAs related to lipid metabolism and known longevity pathway correlates with subsequent life expectancy. **A.** Survivorship curves and imaging of five of six different biological replicates of LL vs. SL worms that were used for RNA-Seq analysis (Replicate 2 shown in Fig 1). **B–E.** Validation of gene expression changes in LL vs. SL worms in RNA-Seq. qRT-PCR of metabolic genes B. *fat-7*; C. *acdh-1*; D. *acdh-2*; and E. *gst-10* in the SL and LL worm sub-populations at Day 6 (****P ≤ 0.0001, ***P ≤ 0.001, **P ≤ 0.01, *P ≤ 0.05; ns *P* > 0.05). *P*-values calculated with unpaired, two-tailed Welch’s *t* test. qRT-PCR data are mean + s.e.m. of 3 biological replicates. **F.** WormCat visualization of categories enriched in genes that exhibit differential local splicing events in LL vs. SL worms. Data underlying the graphs in this figure can be found in S5 Data.
(TIF)

S2 Fig Specificity of RNAi to REPO-1. **A.** Schematic representing the position of genes *repo-1* and *rbm-34* in the *Caenorhabditis elegans* genome operon CEOP4488 and the target sequences of the old *repo-1* RNAi from the Ahringer library (*repo-1*_Ahringer_ RNAi) and the newly constructed *repo-1*_FL_, *repo-1*_short_ RNAi, and *rbm-34*_FL_ RNAi. Approximate positions of the qRT-PCR probes are marked. **B.** *repo-1*_Ahringer_ RNAi knocks down both *repo-1* and *rbm-34*. qRT-PCR of *repo-1* and *rbm-34* expression in Day 1 wild-type worms on control and *repo-1*_Ahringer_ RNAi from hatch. **C.** qRT-PCR of *repo-1* and *rbm-34* expression in Day 1 wild-type worms fed with control, *repo-1*_FL_, *repo-1*_short_ and *rbm-34*_FL_ RNAi from hatch (****P ≤ 0.0001, ***P ≤ 0.001, **P ≤ 0.01, *P ≤ 0.05; ns *P* > 0.05). *P*-values calculated with unpaired, two-tailed Welch’s *t* test. qRT-PCR data are mean + s.e.m. of 3 biological replicates. Increased signal of *repo-1* and *rbm-34* on treatment with *repo-1*_FL_ and *rbm-34*_FL_ RNAi, respectively, is likely due to non-specific signals from the siRNAs produced by bacteria that are ingested by the worms. Note that *repo-1*_short_ RNAi is able to knockdown *repo-1* with equal efficiency as *repo-1*_Ahringer_ RNAi but has no effect on *rbm-34* expression. **D–G.** Knockdown of *repo-1* and not *rbm-34* blocks life span extension in a C. elegans model of longevity. Survivorship of wild-type (WT) and *isp-1(qm150)* worms with or without D. *repo-1*_Ahringer_ (*P* = 0.5434), E. *repo-1*_FL_ (*P* = 0.1228), F. *repo-1*_short_ RNAi (*P* = 0.0006, shorter-lived), and G. *rbm-34*_FL_ RNAi (*P* ≤ 0.0001) (*P*-values are comparing wild-type RNAi vs. *isp-1(qm150)* RNAi in each case). Data underlying the graphs in this figure can be found in S6 Data.
(TIF)

S3 Fig The effect of loss of REPO-1 and SFA-1 is conserved across other mutants in the different longevity pathways. **A.** Effect of loss of REPO-1 is conserved across other mutants of the TORC1 pathway. Survivorship of wild-type (WT) and *rsks-1(ok1255)* −/+ *repo-1* RNAi (*P = 0.0803,* wild-type N2 *repo-1* RNAi vs. *rsks-1(ok1255) repo-1* RNAi, 2 replicates). RNAi was administered from hatch. **B–D.** Effect of loss of REPO-1 is conserved across other mutants of the ETC pathway. **B.** Survivorship of wild-type (WT) and *isp-1(qm50)* on *repo-1* RNAi *(P < 0.0001,* wild-type N2 *repo-1* RNAi vs. *isp-1(qm50) repo-1* RNAi, 2 replicates). RNAi was administered from hatch. **C.** Survivorship of WT and *nuo-6(qm200)* on *repo-1* RNAi (*P = 0.9116,* wild-type N2 *repo-1* RNAi vs. *nuo-6(qm200) repo-1* RNAi, 2 replicates). RNAi was administered from hatch. **D.** Survivorship of WT and *nuo-6(qm200)* on *sfa-1* RNAi (*P < 0.0001,* wild-type N2 *sfa-1* RNAi vs. *nuo-6(qm200) sfa-1* RNAi, 2 replicates). RNAi was administered from hatch. **E.** Effect of loss of REPO-1 is conserved across other mutants of the rIIS pathway. Survivorship of wild-type (WT) and *daf-2(e1370) −/+ repo-1* RNAi (*P < 0.0001,* wild-type N2 *repo-1* RNAi vs. *daf-2(e1370) repo-1* RNAi, 2 replicates). RNAi was administered from hatch. **F, G.** Survivorship of wild-type (WT) and DAF-16a/b over-expressor worms (*daf-16p*::DAF16a/b::GFP) *−/+ repo-1* RNAi (*P* = 0.8994) and *sfa-1* RNAi (*P* = 0.8718) (*P*-values are comparing WT on RNAi vs. mutant on RNAi, 2 replicates). RNAi was administered from hatch. **H.** REPO-1 is knocked down in different mutants with equal efficiency. qPCR showing ~50% knockdown of *repo-1* in different longevity mutants fed with empty vector and *repo-1* RNAi from hatch and collected at Day 1 of adulthood (****P ≤ 0.0001, ***P ≤ 0.001, **P ≤ 0.01, *P ≤ 0.05; ns *P* > 0.05). *P*-values calculated with unpaired, two-tailed Welch’s *t* test. qRT–PCR data are mean + s.e.m. of 3 biological replica*t*es. **I.** Principal Component Analysis of RNA Seq Samples in different longevity mutants −/+ *repo-1* RNAi. Data underlying the graphs in this figure can be found in S6 Data.
(TIF)

S4 Fig Loss of REPO-1 specifically affects lipid metabolism in splicing factor-sensitive longevity pathways. **A.** Expression of *fat-3* by qRT-PCR in wild-type and different mutants −/+ *repo-1* and *sfa-1* RNAi at Day 1. **B.** Normalized counts of *fat-3* transcript in RNA-Seq of wild-type and different mutants −/+ *repo-1* RNAi at Day 1. **C.** Expression of *cpt-5* by qRT-PCR in wild-type and different mutants −/+ * repo-1* and *sfa-1* RNAi at Day 1. **D.** Normalized counts of *cpt-5* transcript in RNA-Seq of wild-type and different mutants −/+ *repo-1* RNAi at Day 1. (****P ≤ 0.0001, ***P ≤ 0.001, **P ≤ 0.01, *P ≤ 0.05; ns *P* > 0.05). *P*-values calculated with unpaired, two-tailed Welch’s *t* test. RNA-seq data are mean + s.e.m. of normalized read counts of 4 biological replicates. qRT-PCR data are mean + s.e.m. of 3 biological replicates. **E.** Schematic representing eCLIP in worms. **F.** Venn diagram displaying the overlap of RNA targets with enrichment >2-fold (log2 fold change >1, IP vs. Input) in SFA-1 and REPO-1. **G.** Validation of *tos-1* as a target of SFA-1 and REPO-1. Semi-quantitative PCR showing differential isoforms of *tos-1* in Day 1 worms on loss of *repo-1* and *sfa-1* using RNAi. Red arrow marks the appearance of a different isoform of *tos-1* on loss of *repo-1* and *sfa-1*. Data underlying the graphs in this figure can be found in S7 Data. The data plotted in panels B and D were taken from values in S5 Table.
(TIF)

S5 Fig Loss of REPO-1 specifically changes the lipidome of splicing factor-sensitive pathways. **A.** Principal component analysis (PCA) of Median Quantitation of Lipid Species across strains and conditions. Each point represents a biological sample, colored according to experimental condition. The ellipses represent 95% confidence intervals for each group**. B.** Boxplot of total triacylglycerol (TG) abundance (nmol/mg of protein) in each experimental condition. We performed Wilcoxon test between Control and repo-1 RNAi in each strain. Only *raga-1* control vs. raga-1 *repo-1* RNAi was significant (fold change = 0.59, *p*-value = 0.02). **C.** Volcano plots for each strain comparing control vs. *repo-1* RNAi. Groups were compared with *t* test, followed by Benjamini–Horchberg correction for multiple comparisons. Significant changes were defined as log fold change >1 or < 1 and *p*-value <0.05. Significant changes are highlighted in red and listed in the table. Data underlying the graphs in this figure can be found in S7 Data.
(TIF)

S6 Fig REPO-1 is expressed at elevated levels in early larval stages of worm development and might be crucial for its function in this time window. **A.** Fluorescent microscope images of worms with CRISPR tagged REPO-1::GFP and CRISPR tagged SFA-1::wrmScarlet at Day 1. Worms imaged at 20× magnification. White arrows mark the presence of REPO-1 and absence of SFA-1 in early embryos. **B.** Fluorescent microscope images of CRISPR tagged REPO-1 fused to GFP and CRISPR tagged SFA-1 fused to wrmScarlet from the egg stage to Day 1 of adulthood. **C.** REPO-1 and SFA-1 mRNA levels do not change with age. Normalized counts of *repo-1* and *sfa-1* transcripts at Day 3 and Day 15 in WT and *eat-2(ad1116)* worms. Transcript counts obtained from previously published RNA-Seq data (Heintz and colleagues. Nature 2017 [6]). **D, E.** REPO-1 protein levels do not change with age. **D.** Western blotting of CRISPR tagged endogenous 3XFLAG::REPO-1 worms at Day1, Day 5, Day 10, and Day 15 of adulthood. Blots probed with 3XFLAG and actin antibodies. Ponceau staining of the blot shows equal loading. **E.** Western blotting of CRISPR-tagged endogenous 3XFLAG::REPO-1 worms at L1–L2, L3–L4, Day 1, and Day 15 of adulthood. Blots probed with 3XFLAG and actin antibodies. Ponceau staining of the blot shows equal loading. **F.** Quantification of 3XFLAG:REPO-1 normalized to actin. Blots quantified using ImageJ and represented as percent of expression at Day 1 of adulthood. **G, H.** Adult-onset RNAi efficiently knocks down REPO-1 and SFA-1 comparable to RNAi from hatch. Western blotting of CRISPR tagged endogenous **G.** 3XFLAG::REPO-1 worms −/+ *repo-1* RNAi; **I.** 3XFLAG::SFA-1 worms −/+ *sfa-1* RNAi from hatch or Day 1 of adulthood (D1-onset). Samples collected on Day 1, Day 2, and Day 3 to measure efficiency of knockdown. Lysates probed for 3XFLAG as a readout of REPO-1/SFA-1 and actin as loading control. Quantification of knockdown of **H.** REPO-1 and **J.** SFA-1 normalized to actin. Blots quantified using ImageJ and represented as percent of RNAi untreated control. (****P ≤ 0.0001, ***P ≤ 0.001, **P ≤ 0.01, *P ≤ 0.05; ns *P* > 0.05). *P*-values calculated with unpaired, two-tailed Welch’s *t* test. Data underlying the graphs in this figure can be found in S7 Data.
(TIF)

S1 Table This table contains normalized counts of all genes obtained from RNA-Seq of worms with long life expectancy (LL) and short life expectancy (SL). (XLSX)

S2 Table This table contains differentially expressed genes from RNA-Seq of worms with long life expectancy (LL) vs. short life expectancy (SL) and the WormCat functional categories of the genes. **A.** all genes from differential expression analysis; **B.** significant genes only (log fold change > 1.5 and padj < 0.01); **C.** significant downregulated genes; **D.** significant upregulated genes; **E.** WormCat categories of significantly downregulated in LL vs. SL; **F.** WormCat categories of significantly upregulated in LL vs. SL.
(XLSX)

S3 Table This table contains differential splice isoforms from RNA-Seq of worms with long life expectancy (LL) and short life expectancy (SL). **A.** LL vs. SL significant isoforms (*p*-value < 0.05); **B.** LL vs. SL all isoforms.
(XLSX)

S4 Table This table contains WormCat functional categories of genes that exhibit differential splice isoforms in LL vs. SL. (XLSX)

S5 Table This table contains normalized counts of all genes obtained from RNA-Seq of wild-type and longevity mutants *eat-2(ad1116)*, *raga-1(ok386)*, *clk-1(qm30),* and *age-1(hx546)* without and with *repo-1* RNAi. (XLSB)

S6 Table This table contains analysis of the *repo-1* knockdown effect in the *eat-2(ad1116)*, *raga-1(ok386), clk-1(qm30)*, and *age-(hx546)* mutants. It contains the full list of genes from the analysis in each mutant and genes that respond significantly differently to *repo-1* RNAi using an adjusted p-value cut-off of 0.05. The table also contains a list of the shared 620 genes that respond differentially on loss of *repo-1* in *eat-2(ad1116), raga-1(ok386)*, and *clk-1(qm30)* compared to wild-type N2 worms.
(XLSX)

S7 Table This table contains WormCat functional categories of the 620 genes that respond differentially on loss of *repo-1* in *eat-2(ad1116), raga-1(ok386)*, and *clk-1(qm30)* compared to wild-type N2 worms. (XLSX)

S8 Table This table contains the normalized abundance of all lipid species obtained from lipidomics of wild-type and longevity mutants *eat-2*(ad1116), *raga-1*(ok386), *clk-1*(qm30), and *age-1*(hx546) without and with *repo-1* RNAi. (XLSX)

S9 Table This table contains the eCLIP targets of SF1 and SF3A2 in MEFs. For both S1 and SF3A2, there are lists for—**A.** all peaks from Replicate 1; **B.** significant peaks from Replicate 1 [log2 fold change > 2, −log10(*P*-value) > 2]; **C.** all peaks from Replicate 2; **D.** significant peaks from Replicate 2 [log2 fold change > 2, −log10(*P*-value) > 2]. This table also contains the reproducible SF1 and SF3A2 gene targets from both the runs and the shared targets.
(XLSX)

S10 Table This table contains the eCLIP targets of SFA-1 and REPO-1 in *Caenorhabditis elegans*. **A.** Significant peaks of SFA-1 [log2 fold change > 1, −log10(*P*-value) > 1]; **B.** significant peaks of REPO-1 [log2 fold change > 1, −log10(*P*-value) > 1]; **C.** unique and shared targets of SFA-1 and REPO-1.
(XLSX)

S11 Table This table contains a summary of data from all survival analysis—strains, genotypes, treatments, median life span, *p*-values. All replicates included.
(XLSX)

S1 Data Data supplement for Fig 1. (XLSX)

S2 Data Data supplement for Fig 2. (XLSX)

S3 Data Data supplement for Fig 3. (XLSX)

S4 Data Data supplement for Fig 4. (XLSX)

S5 Data Data supplement for S1 Fig. (XLSX)

S6 Data Data supplement for S2 and S3 Figs. (XLSX)

S7 Data Data supplement for S4, S5, and S6 Figs. (XLSX)

S1 Raw Images Data supplement for Western Blot Full Gel Images. (PDF)

S1 Text Supplementary material. (DOCX)

## Abbreviations

ACC1: Acetyl CoA Carboxylase 1
DR: dietary restriction
eCLIP: enhanced cross-linking immunoprecipitation
IIS: insulin-like growth factor signalling
LL: long life
MEF: mouse embryonic fibroblast
REPO-1: Reversed Polarity-1
SFA-1: Splicing Factor 1
SL: short life
SRS: stimulated Raman scattering
WT: wild-type
