# Supplementary figures and images for: The efficacy of longevity interventions in Caenorhabditis elegans is determined by the early life activity of RNA splicing factors
Source: PLoS Biol. 2025 Nov 21;23(11):e3003504. doi: 10.1371/journal.pbio.3003504 (PMC12671814; doi:10.1371/journal.pbio.3003504)

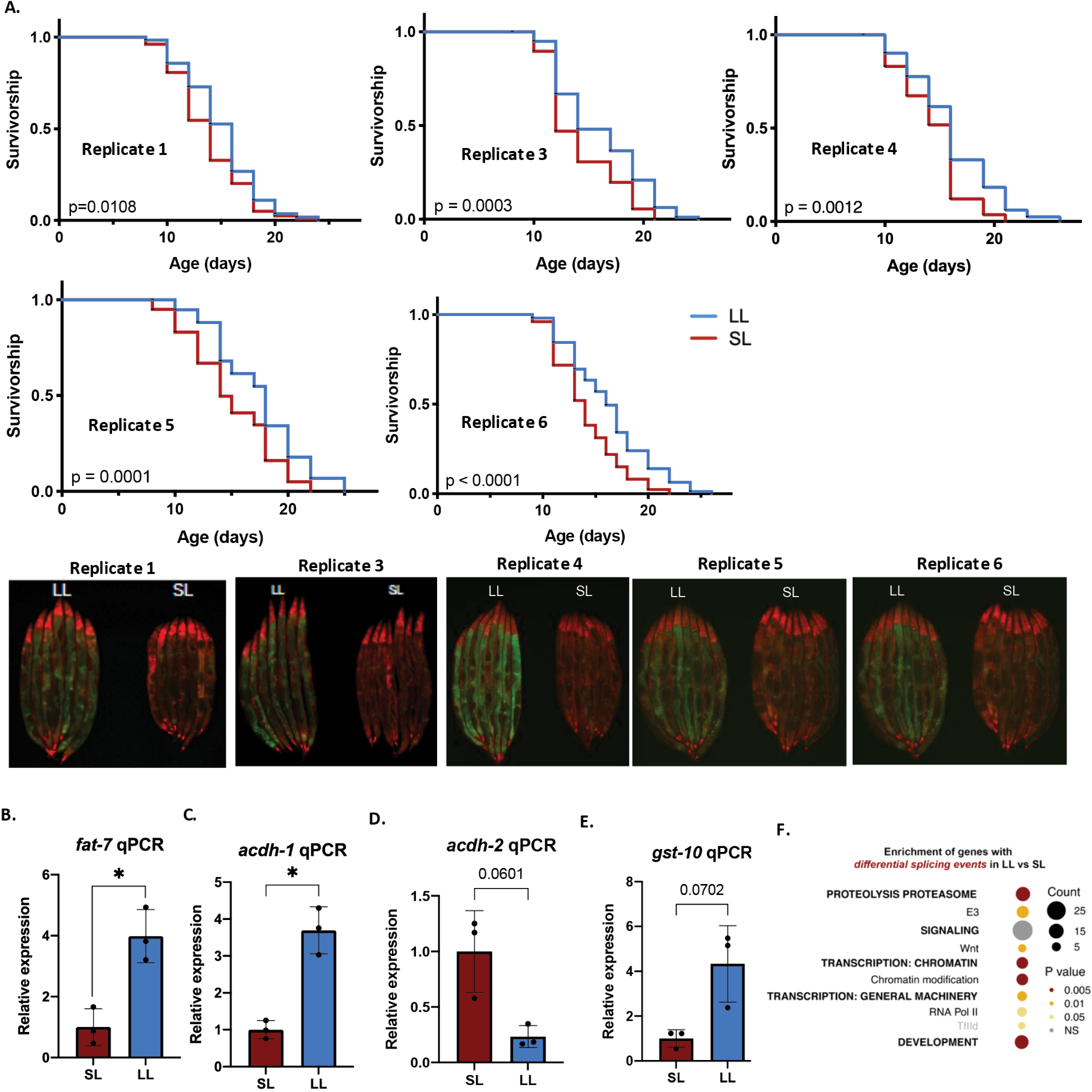

Supplement: S1 Fig — A. Survivorship curves and imaging of five of six different biological replicates of LL vs. SL worms that were used for RNA-Seq analysis (Replicate 2 shown in Fig 1). B–E. Validation of gene expression changes in LL vs. SL worms in RNA-Seq. qRT-PCR of metabolic genes B. fat-7; C. acdh-1; D. acdh-2; and E. gst-10 in the SL and LL worm sub-populations at Day 6 (****P ≤ 0.0001, ***P ≤ 0.001, **P ≤ 0.01, *P ≤ 0.05; ns P > 0.05). P-values calculated with unpaired, two-tailed Welch’s t test. qRT-PCR data are mean + s.e.m. of 3 biological replicates. F. WormCat visualization of categories enriched in genes that exhibit differential local splicing events in LL vs. SL worms. Data underlying the graphs in this figure can be found in S5 Data. (TIF) [file pbio.3003504.s001.tif]

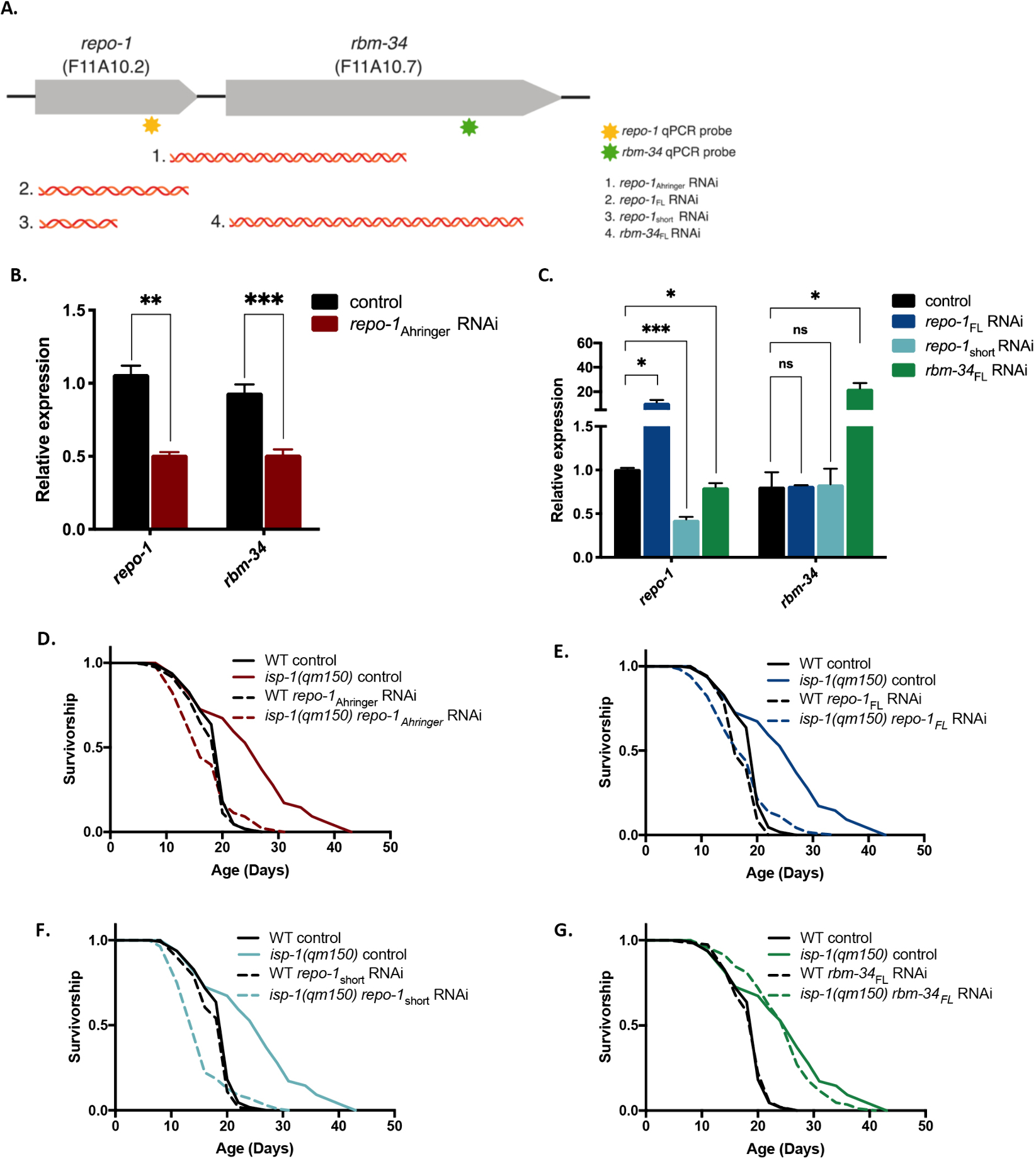

Supplement: S2 Fig — A. Schematic representing the position of genes repo-1 and rbm-34 in the Caenorhabditis elegans genome operon CEOP4488 and the target sequences of the old repo-1 RNAi from the Ahringer library (repo-1Ahringer RNAi) and the newly constructed repo-1FL, repo-1short RNAi, and rbm-34FL RNAi. Approximate positions of the qRT-PCR probes are marked. B. repo-1Ahringer RNAi knocks down both repo-1 and rbm-34. qRT-PCR of repo-1 and rbm-34 expression in Day 1 wild-type worms on control and repo-1Ahringer RNAi from hatch. C. qRT-PCR of repo-1 and rbm-34 expression in Day 1 wild-type worms fed with control, repo-1FL, repo-1short and rbm-34FL RNAi from hatch (****P ≤ 0.0001, ***P ≤ 0.001, **P ≤ 0.01, *P ≤ 0.05; ns P > 0.05). P-values calculated with unpaired, two-tailed Welch’s t test. qRT-PCR data are mean + s.e.m. of 3 biological replicates. Increased signal of repo-1 and rbm-34 on treatment with repo-1FL and rbm-34FL RNAi, respectively, is likely due to non-specific signals from the siRNAs produced by bacteria that are ingested by the worms. Note that repo-1short RNAi is able to knockdown repo-1 with equal efficiency as repo-1Ahringer RNAi but has no effect on rbm-34 expression. D–G. Knockdown of repo-1 and not rbm-34 blocks life span extension in a C. elegans model of longevity. Survivorship of wild-type (WT) and isp-1(qm150) worms with or without D. repo-1Ahringer (P = 0.5434), E. repo-1FL (P = 0.1228), F. repo-1short RNAi (P = 0.0006, shorter-lived), and G. rbm-34FL RNAi (P ≤ 0.0001) (P-values are comparing wild-type RNAi vs. isp-1(qm150) RNAi in each case). Data underlying the graphs in this figure can be found in S6 Data. (TIF) [file pbio.3003504.s002.tif]

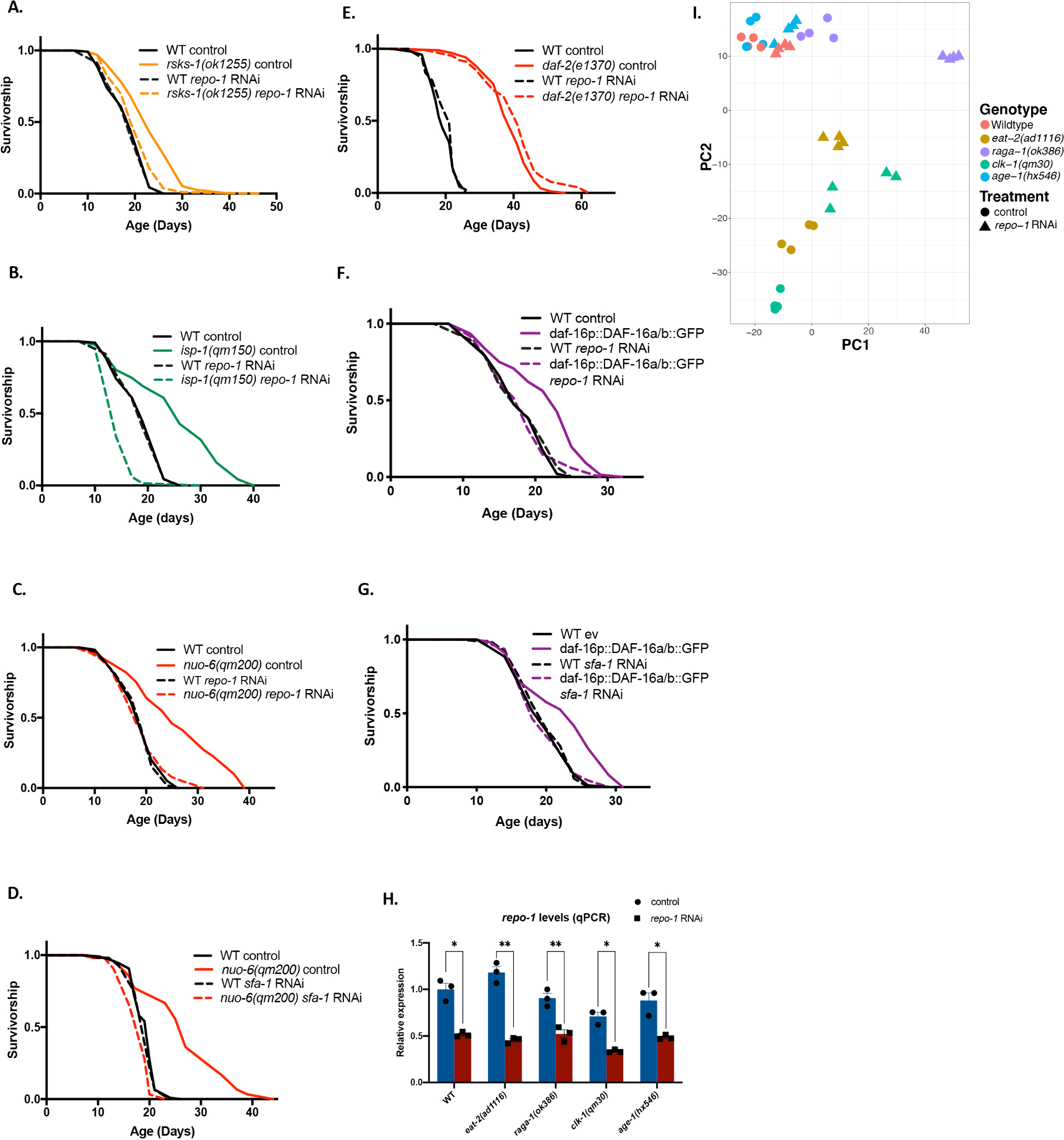

Supplement: S3 Fig — A. Effect of loss of REPO-1 is conserved across other mutants of the TORC1 pathway. Survivorship of wild-type (WT) and rsks-1(ok1255) −/+ repo-1 RNAi (P = 0.0803, wild-type N2 repo-1 RNAi vs. rsks-1(ok1255) repo-1 RNAi, 2 replicates). RNAi was administered from hatch. B–D. Effect of loss of REPO-1 is conserved across other mutants of the ETC pathway. B. Survivorship of wild-type (WT) and isp-1(qm50) on repo-1 RNAi (P < 0.0001, wild-type N2 repo-1 RNAi vs. isp-1(qm50) repo-1 RNAi, 2 replicates). RNAi was administered from hatch. C. Survivorship of WT and nuo-6(qm200) on repo-1 RNAi (P = 0.9116, wild-type N2 repo-1 RNAi vs. nuo-6(qm200) repo-1 RNAi, 2 replicates). RNAi was administered from hatch. D. Survivorship of WT and nuo-6(qm200) on sfa-1 RNAi (P < 0.0001, wild-type N2 sfa-1 RNAi vs. nuo-6(qm200) sfa-1 RNAi, 2 replicates). RNAi was administered from hatch. E. Effect of loss of REPO-1 is conserved across other mutants of the rIIS pathway. Survivorship of wild-type (WT) and daf-2(e1370) −/+ repo-1 RNAi (P < 0.0001, wild-type N2 repo-1 RNAi vs. daf-2(e1370) repo-1 RNAi, 2 replicates). RNAi was administered from hatch. F, G. Survivorship of wild-type (WT) and DAF-16a/b over-expressor worms (daf-16p::DAF16a/b::GFP) −/+ repo-1 RNAi (P = 0.8994) and sfa-1 RNAi (P = 0.8718) (P-values are comparing WT on RNAi vs. mutant on RNAi, 2 replicates). RNAi was administered from hatch. H. REPO-1 is knocked down in different mutants with equal efficiency. qPCR showing ~50% knockdown of repo-1 in different longevity mutants fed with empty vector and repo-1 RNAi from hatch and collected at Day 1 of adulthood (****P ≤ 0.0001, ***P ≤ 0.001, **P ≤ 0.01, *P ≤ 0.05; ns P > 0.05). P-values calculated with unpaired, two-tailed Welch’s t test. qRT–PCR data are mean + s.e.m. of 3 biological replicates. I. Principal Component Analysis of RNA Seq Samples in different longevity mutants −/+ repo-1 RNAi. Data underlying the graphs in this figure can be found in S6 Data. (TIF) [file pbio.3003504.s003.tif]

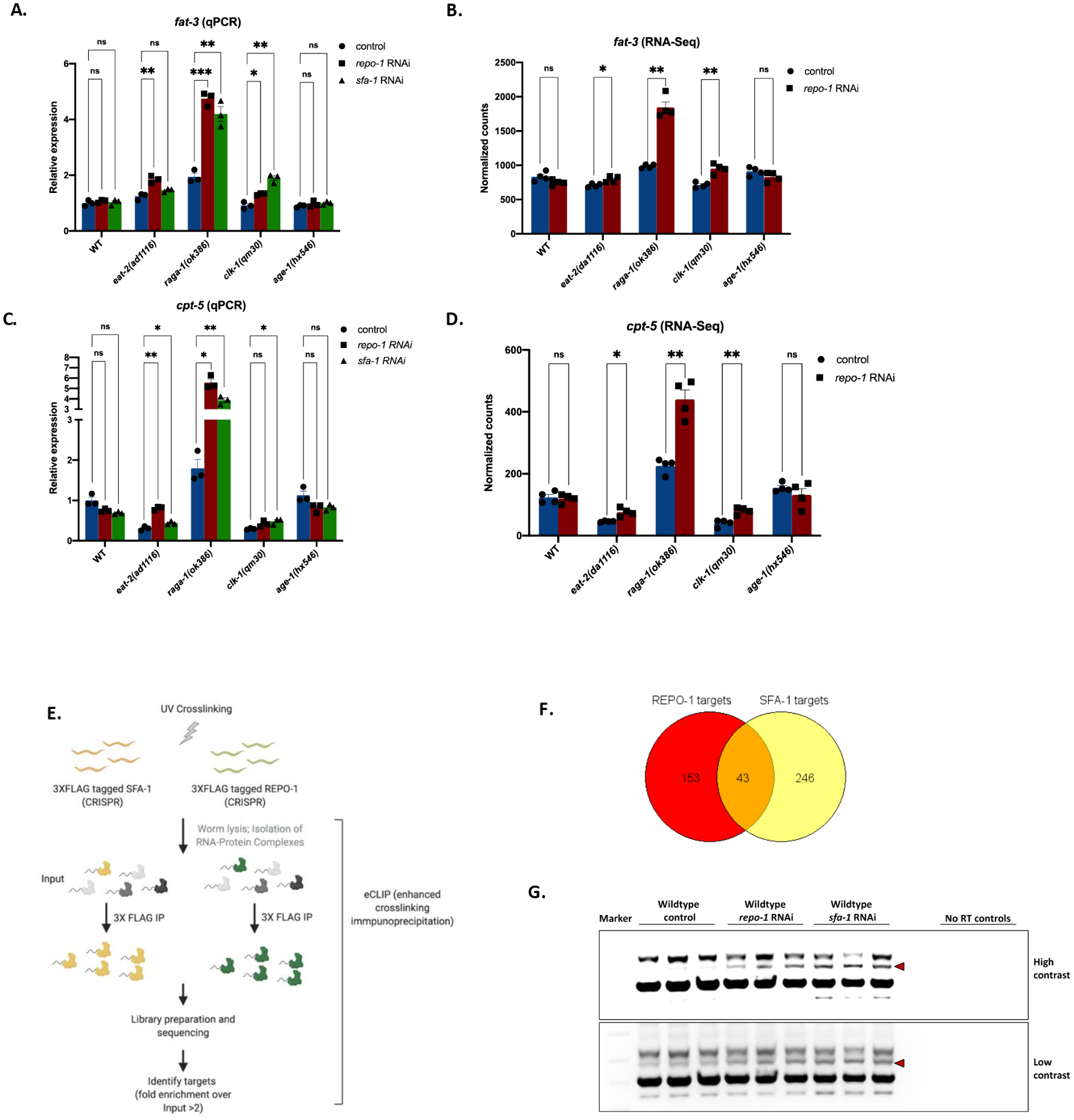

Supplement: S4 Fig — A. Expression of fat-3 by qRT-PCR in wild-type and different mutants −/+ repo-1 and sfa-1 RNAi at Day 1. B. Normalized counts of fat-3 transcript in RNA-Seq of wild-type and different mutants −/+ repo-1 RNAi at Day 1. C. Expression of cpt-5 by qRT-PCR in wild-type and different mutants −/+ repo-1 and sfa-1 RNAi at Day 1. D. Normalized counts of cpt-5 transcript in RNA-Seq of wild-type and different mutants −/+ repo-1 RNAi at Day 1. (****P ≤ 0.0001, ***P ≤ 0.001, **P ≤ 0.01, *P ≤ 0.05; ns P > 0.05). P-values calculated with unpaired, two-tailed Welch’s t test. RNA-seq data are mean + s.e.m. of normalized read counts of 4 biological replicates. qRT-PCR data are mean + s.e.m. of 3 biological replicates. E. Schematic representing eCLIP in worms. F. Venn diagram displaying the overlap of RNA targets with enrichment >2-fold (log2 fold change >1, IP vs. Input) in SFA-1 and REPO-1. G. Validation of tos-1 as a target of SFA-1 and REPO-1. Semi-quantitative PCR showing differential isoforms of tos-1 in Day 1 worms on loss of repo-1 and sfa-1 using RNAi. Red arrow marks the appearance of a different isoform of tos-1 on loss of repo-1 and sfa-1. Data underlying the graphs in this figure can be found in S7 Data. The data plotted in panels B and D were taken from values in S5 Table. (TIF) [file pbio.3003504.s004.tif]

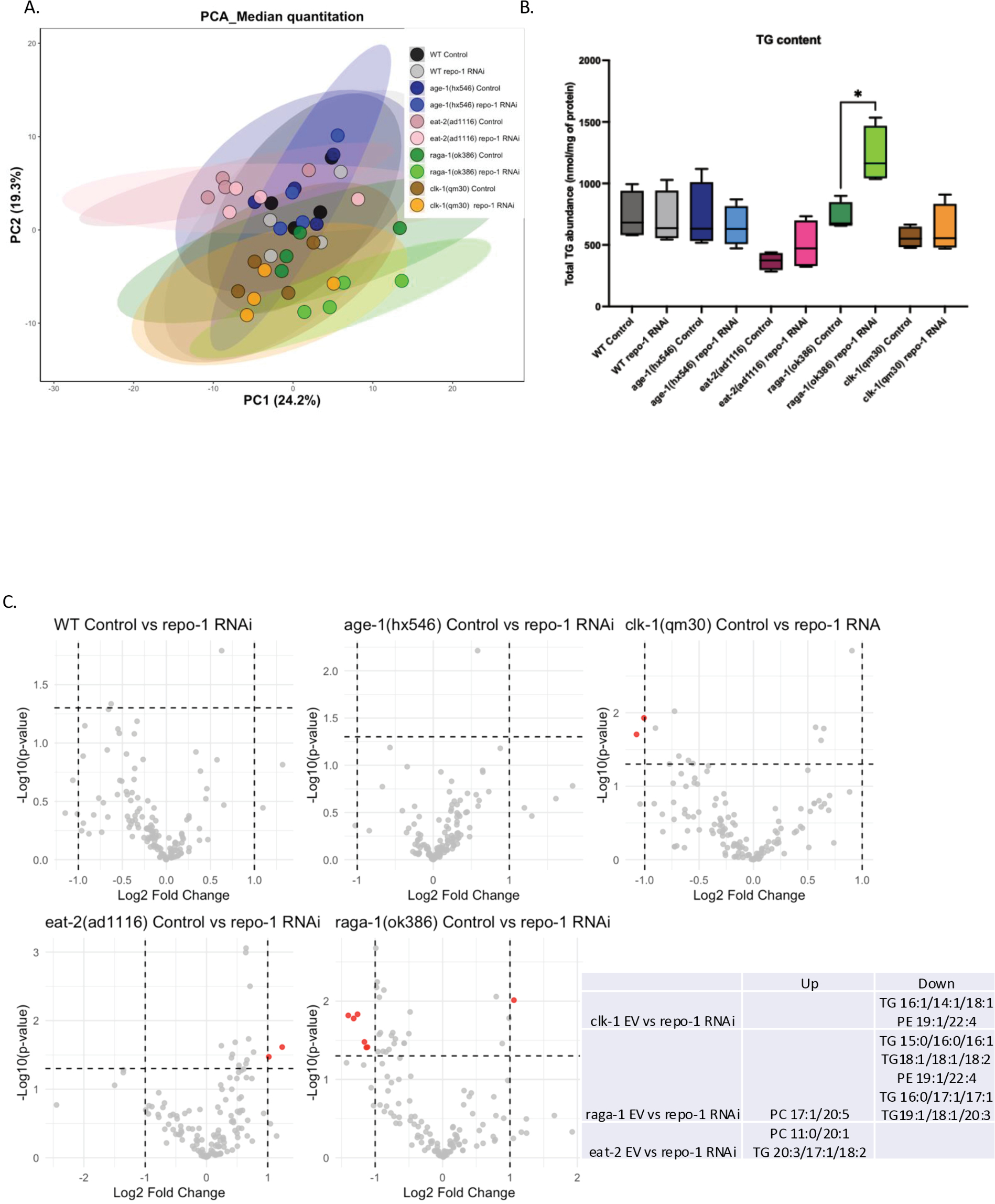

Supplement: S5 Fig — A. Principal component analysis (PCA) of Median Quantitation of Lipid Species across strains and conditions. Each point represents a biological sample, colored according to experimental condition. The ellipses represent 95% confidence intervals for each group. B. Boxplot of total triacylglycerol (TG) abundance (nmol/mg of protein) in each experimental condition. We performed Wilcoxon test between Control and repo-1 RNAi in each strain. Only raga-1 control vs. raga-1 repo-1 RNAi was significant (fold change = 0.59, p-value = 0.02). C. Volcano plots for each strain comparing control vs. repo-1 RNAi. Groups were compared with t test, followed by Benjamini–Horchberg correction for multiple comparisons. Significant changes were defined as log fold change >1 or < 1 and p-value <0.05. Significant changes are highlighted in red and listed in the table. Data underlying the graphs in this figure can be found in S7 Data. (TIF) [file pbio.3003504.s005.tif]

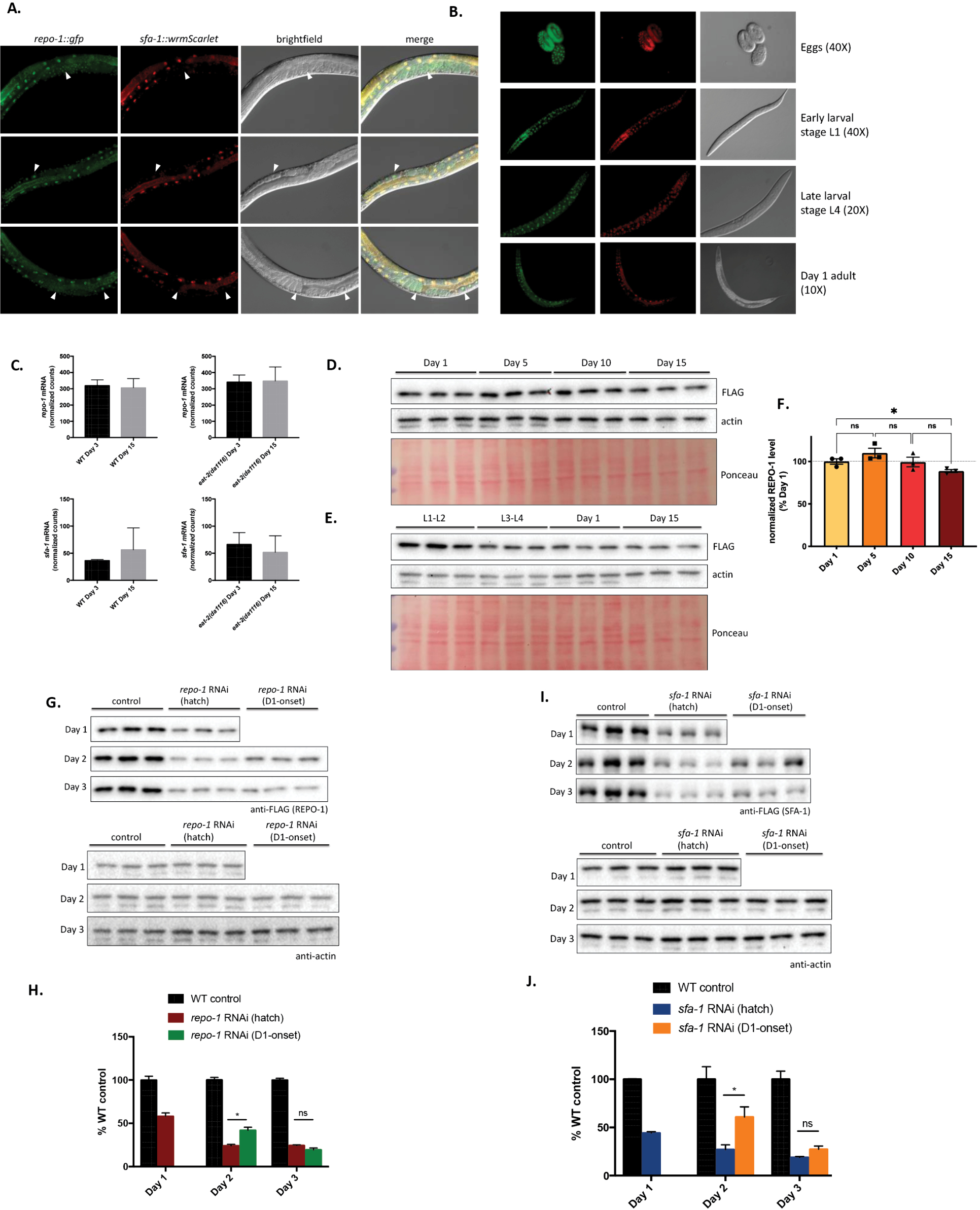

Supplement: S6 Fig — A. Fluorescent microscope images of worms with CRISPR tagged REPO-1::GFP and CRISPR tagged SFA-1::wrmScarlet at Day 1. Worms imaged at 20× magnification. White arrows mark the presence of REPO-1 and absence of SFA-1 in early embryos. B. Fluorescent microscope images of CRISPR tagged REPO-1 fused to GFP and CRISPR tagged SFA-1 fused to wrmScarlet from the egg stage to Day 1 of adulthood. C. REPO-1 and SFA-1 mRNA levels do not change with age. Normalized counts of repo-1 and sfa-1 transcripts at Day 3 and Day 15 in WT and eat-2(ad1116) worms. Transcript counts obtained from previously published RNA-Seq data (Heintz and colleagues. Nature 2017 [6]). D, E. REPO-1 protein levels do not change with age. D. Western blotting of CRISPR tagged endogenous 3XFLAG::REPO-1 worms at Day1, Day 5, Day 10, and Day 15 of adulthood. Blots probed with 3XFLAG and actin antibodies. Ponceau staining of the blot shows equal loading. E. Western blotting of CRISPR-tagged endogenous 3XFLAG::REPO-1 worms at L1–L2, L3–L4, Day 1, and Day 15 of adulthood. Blots probed with 3XFLAG and actin antibodies. Ponceau staining of the blot shows equal loading. F. Quantification of 3XFLAG:REPO-1 normalized to actin. Blots quantified using ImageJ and represented as percent of expression at Day 1 of adulthood. G, H. Adult-onset RNAi efficiently knocks down REPO-1 and SFA-1 comparable to RNAi from hatch. Western blotting of CRISPR tagged endogenous G. 3XFLAG::REPO-1 worms −/+ repo-1 RNAi; I. 3XFLAG::SFA-1 worms −/+ sfa-1 RNAi from hatch or Day 1 of adulthood (D1-onset). Samples collected on Day 1, Day 2, and Day 3 to measure efficiency of knockdown. Lysates probed for 3XFLAG as a readout of REPO-1/SFA-1 and actin as loading control. Quantification of knockdown of H. REPO-1 and J. SFA-1 normalized to actin. Blots quantified using ImageJ and represented as percent of RNAi untreated control. (****P ≤ 0.0001, ***P ≤ 0.001, **P ≤ 0.01, *P ≤ 0.05; ns P > 0.05). P-values calculated with unpaired, two-tailed Welch’s t [file pbio.3003504.s006.tif]

**S4G**

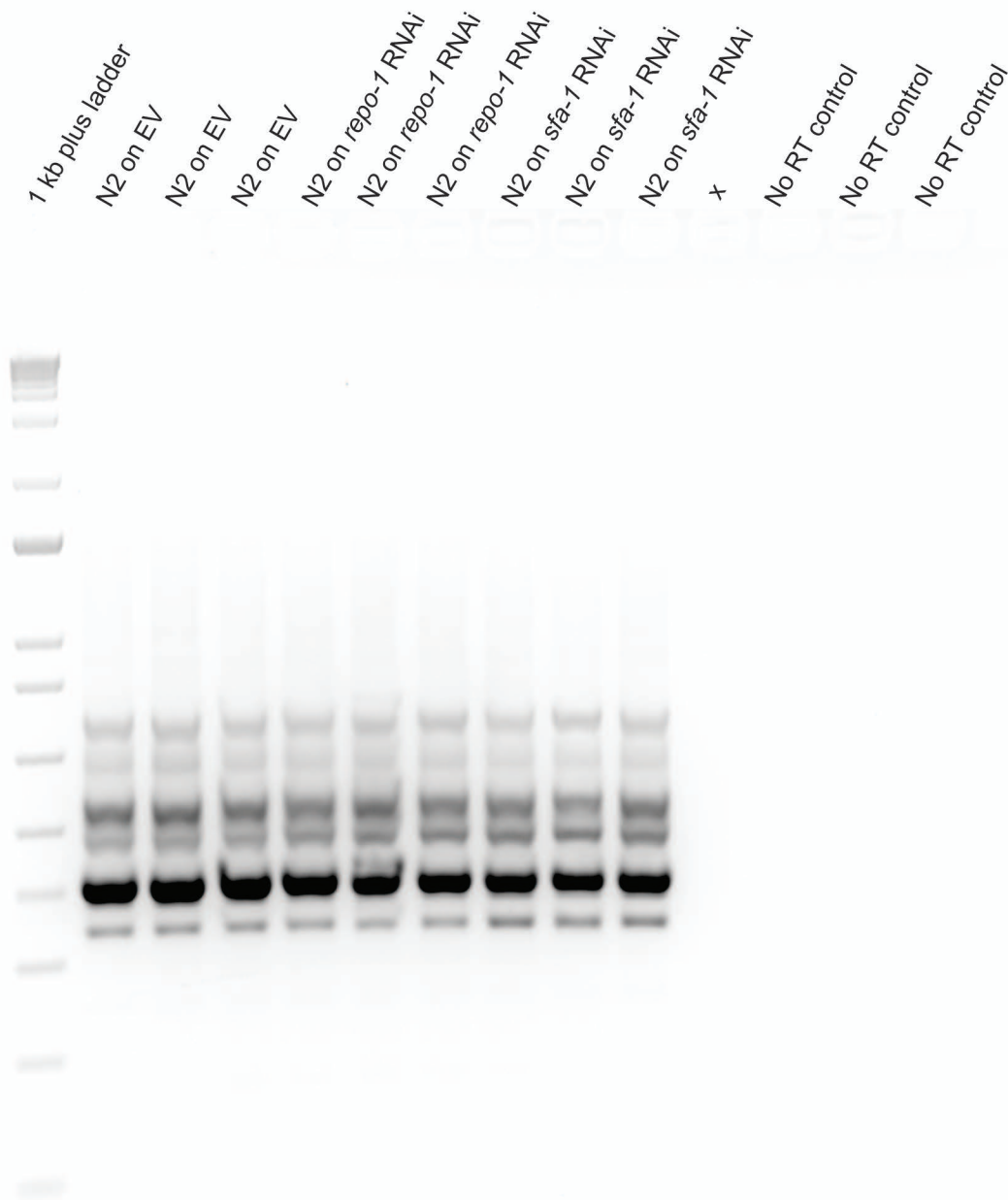

anti-FLAG:

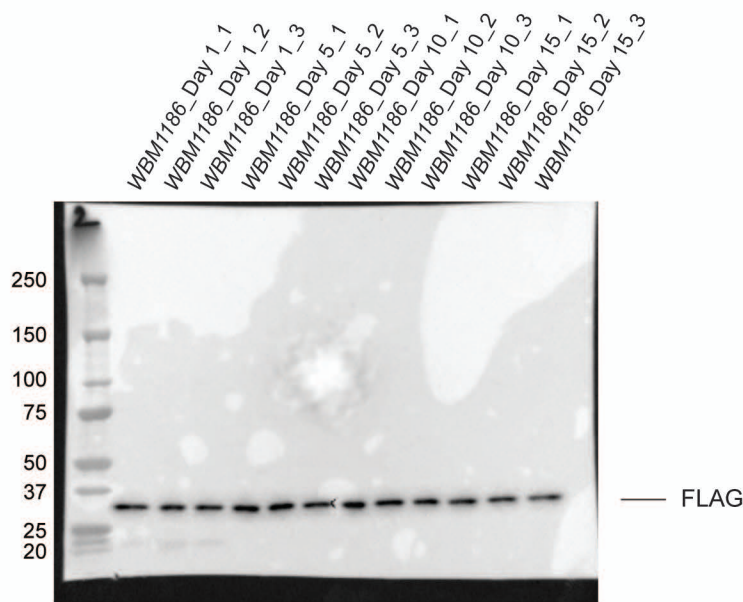

anti-Actin:

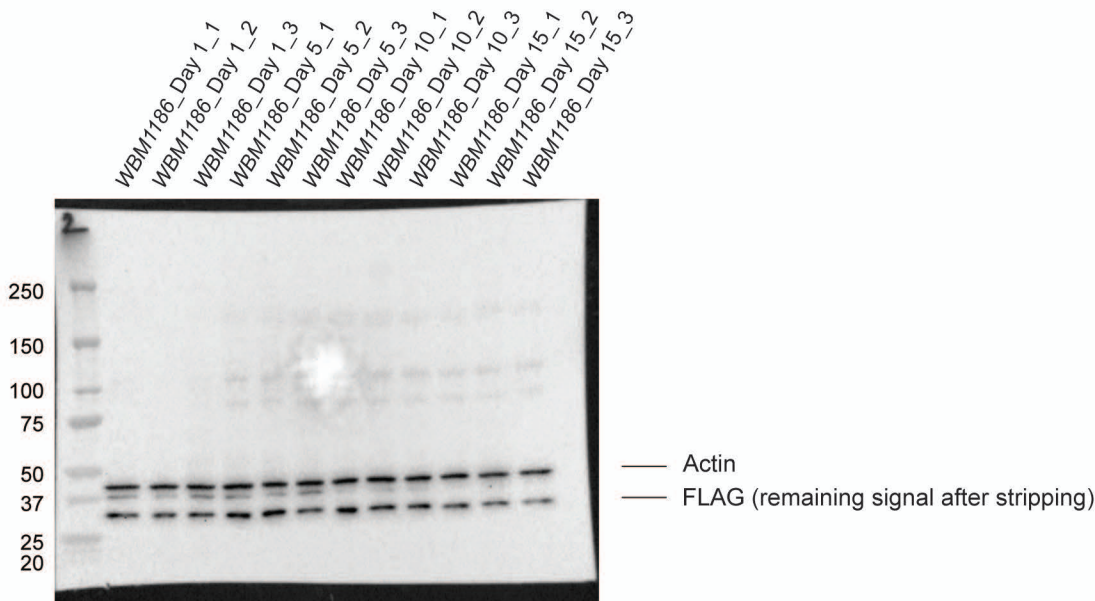

anti-FLAG:

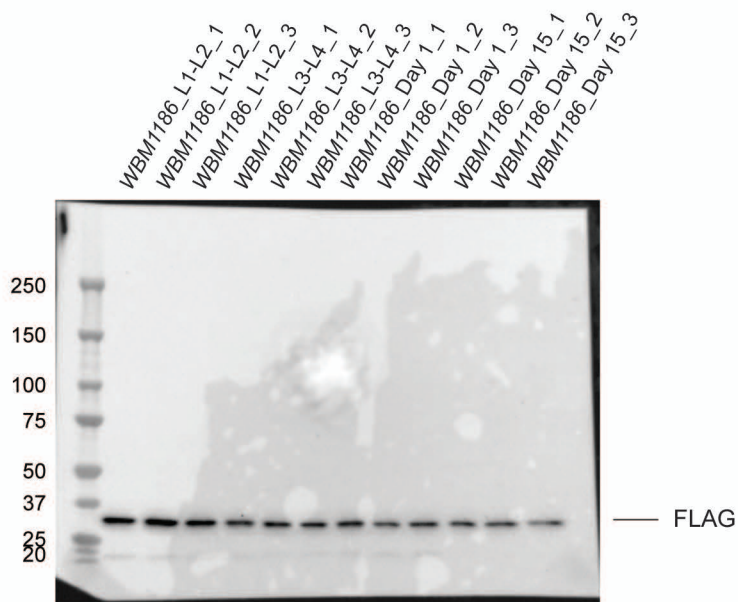

anti-Actin:

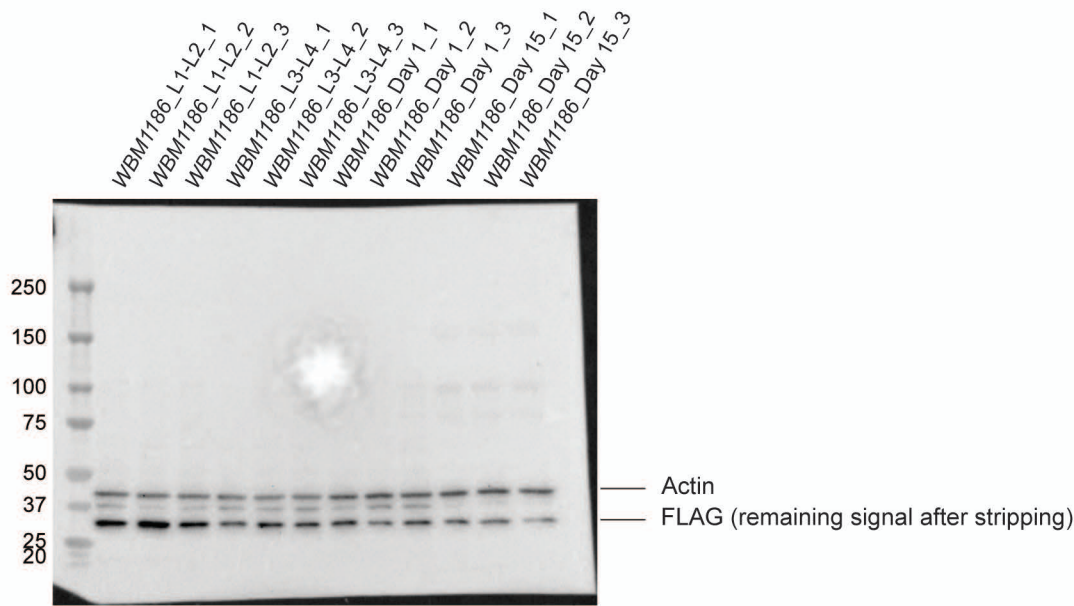

anti-FLAG:

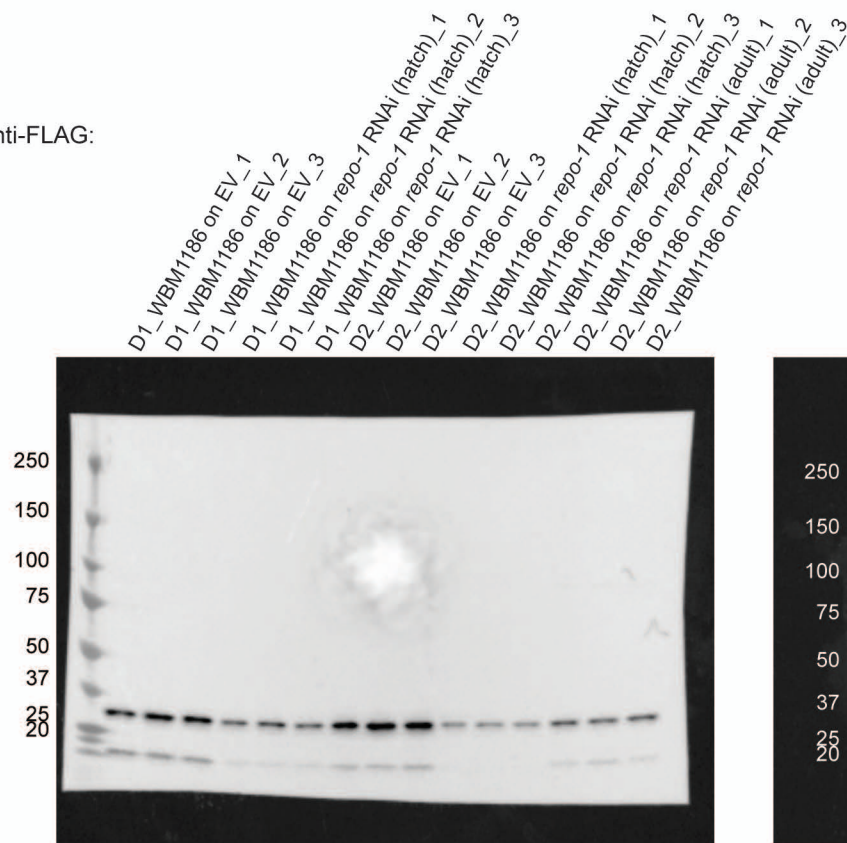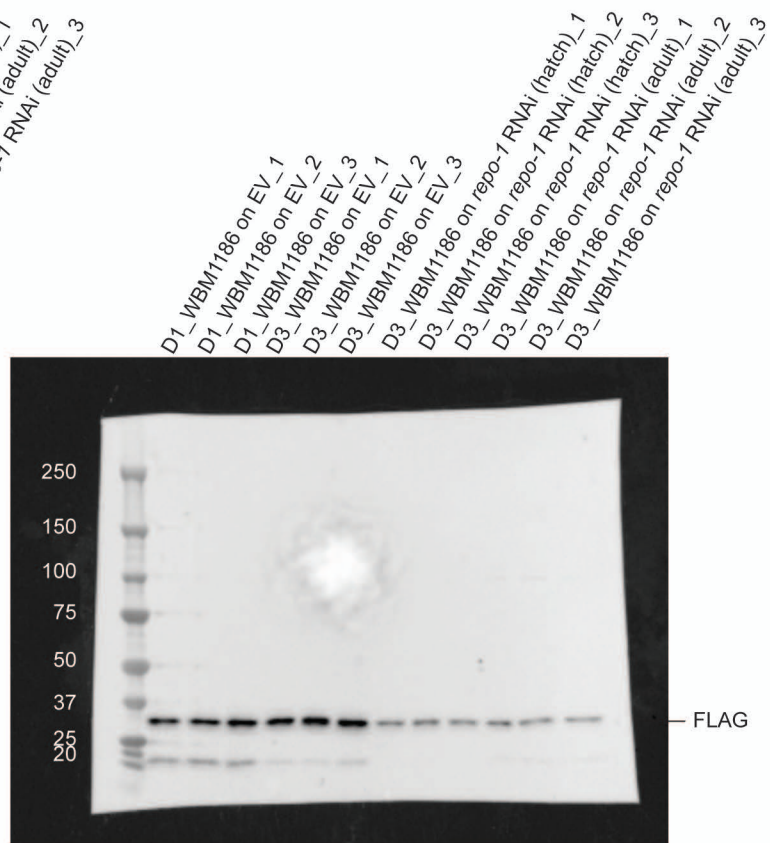

anti-Actin:

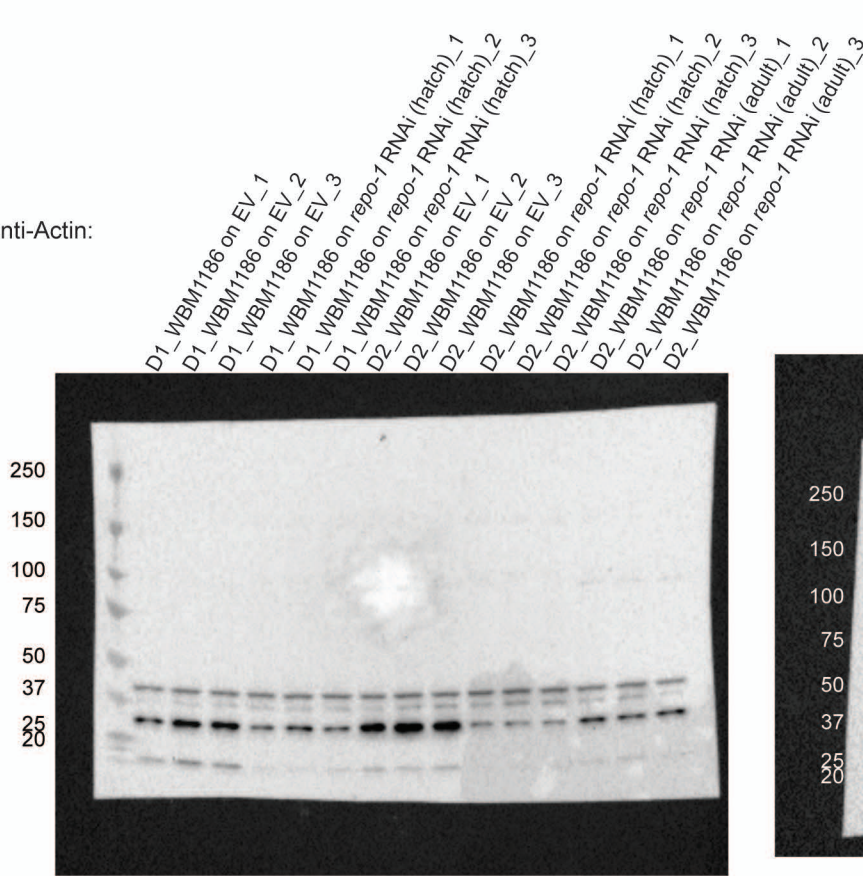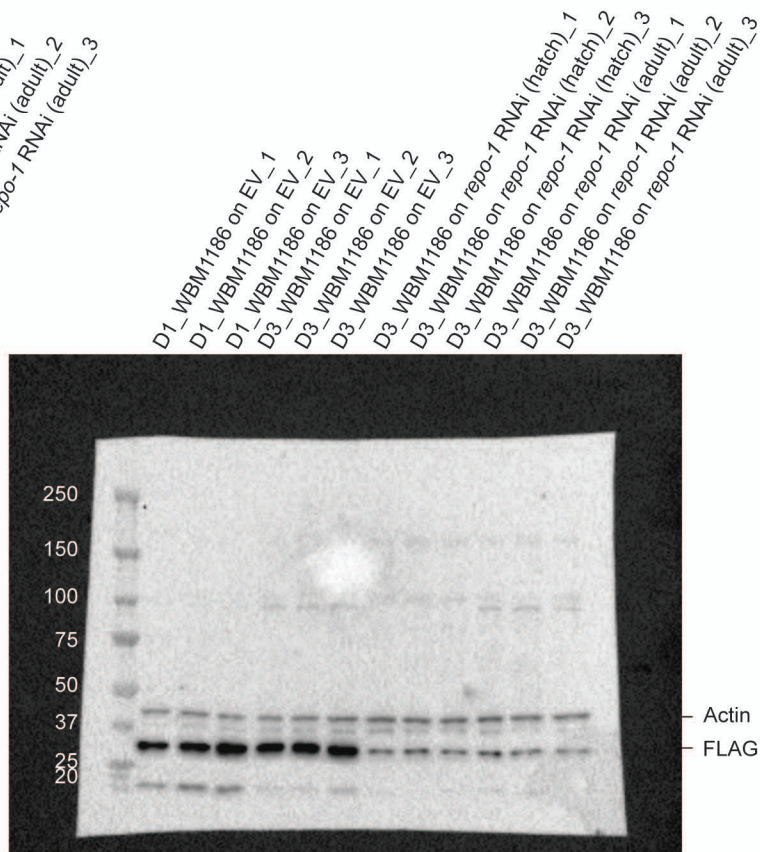

anti-FLAG:

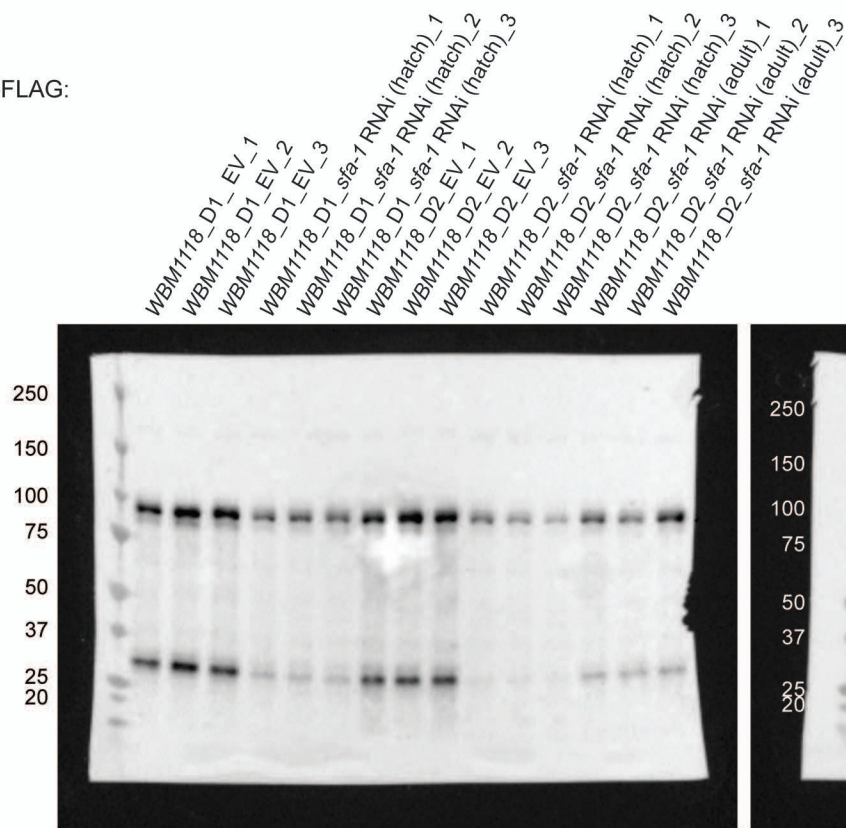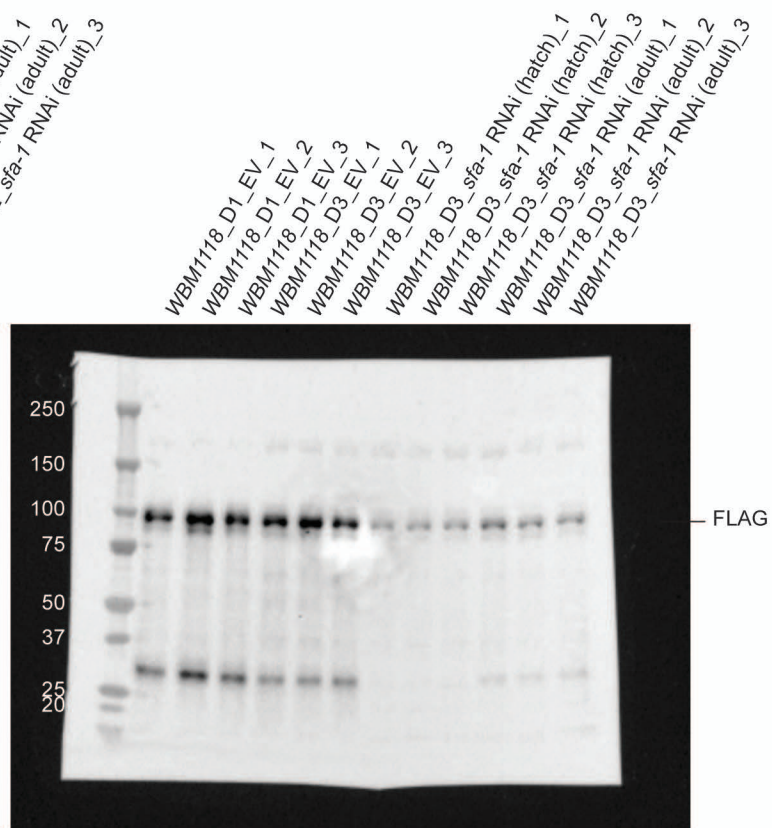

anti-Actin:

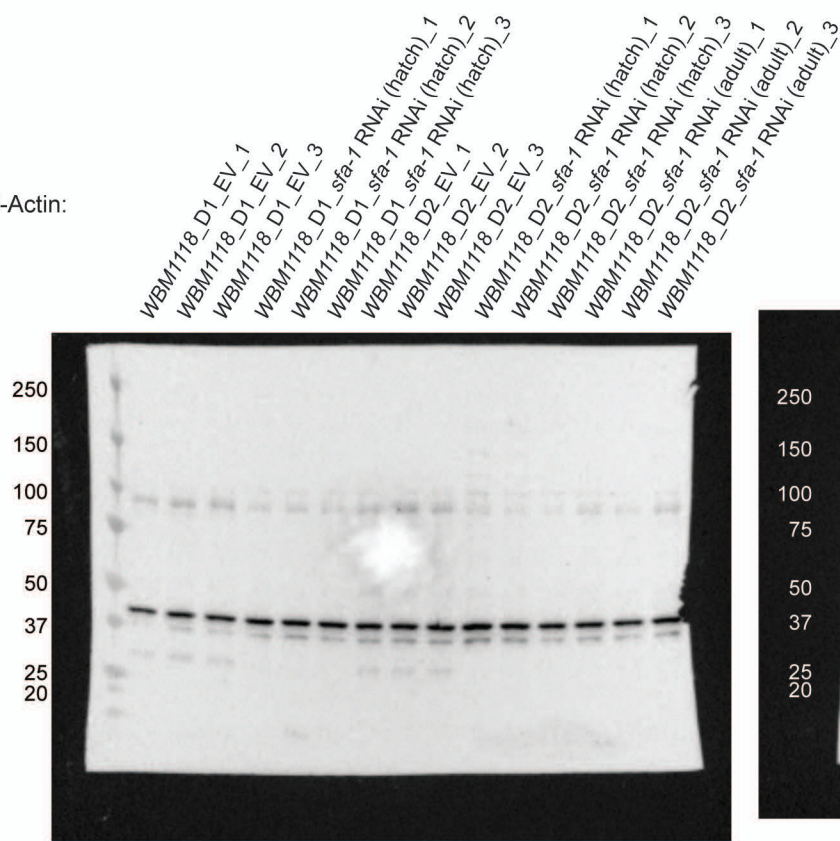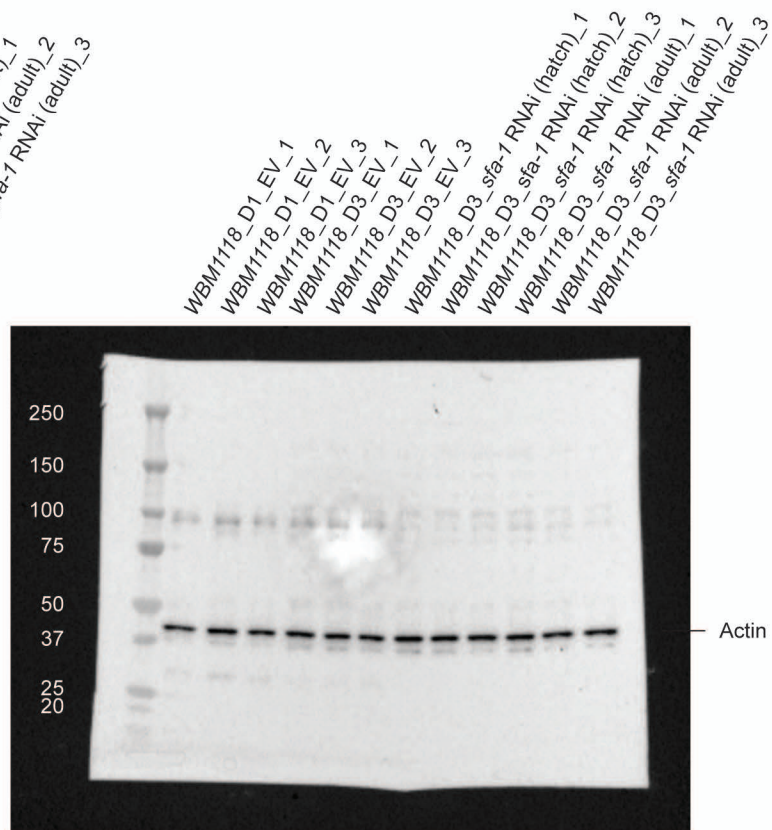

Supplement: S1 Raw Images — (PDF) [file pbio.3003504.s025.pdf]
