# Supplementary material for: The efficacy of longevity interventions in Caenorhabditis elegans is determined by the early life activity of RNA splicing factors
Source: PLoS Biol. 2025 Nov 21;23(11):e3003504. doi: 10.1371/journal.pbio.3003504 (PMC12671814; doi:10.1371/journal.pbio.3003504)
Supplement: S1 Text — (DOCX) [file pbio.3003504.s026.docx]

The efficacy of longevity interventions in *C. elegans*is determined by early

life activity of RNA splicing factors

**Authors:** Sneha Dutta^1#^, Maria Camila Perez Matos^1#^, Caroline Heintz^1^, Ayse Sena Mutlu^2^, Mary Piper^3^, Meeta Mistry^3^, Arpit Sharma^1^, Hannah Smith^1^, Porsha Howell^1^, Rohan Sehgal^1^, Anne Lanjuin^1^, Meng C. Wang^2, 4^, and William B. Mair^1^*

**Affiliations:**

^1^Department of Molecular Metabolism, Harvard T. H. Chan School of Public Health, Harvard University, Boston, Massachusetts 02115, USA.

^2^Huffington Center on Aging, Baylor College of Medicine, Houston, TX, 77030, USA. Howard Hughes Medical Institute, Baylor College of Medicine, Houston, TX 77030, USA.

^3^Harvard Chan Bioinformatics Core, Harvard T. H. Chan School of Public Health, Boston, Massachusetts 02115, USA.

^4^Research Campus, Howard Hughes Medical Institute, Ashburn, VA, USA

**This PDF file includes:**

Materials and Methods

Legends for Supp Tables S1 to S11

Legends for Supporting Data files S1-S7

References for Methods

**MATERIALS AND METHODS**

**Worm strains and culture**

The *C. elegans*strains used for this work are listed below. Worms were routinely grown and maintained on standard nematode growth media (NGM) plates that were seeded with *E. coli* (OP50-1). *E. coli* bacteria were cultured overnight in LB at 37°C, after which 100 µl of liquid culture was seeded on plates to grow for 2 days at room temperature.

| **Strain Name** | **Genotype** | **Notes** | **Source** |
| --- | --- | --- | --- |
| N2 Bristol | Wild type | - | CGC |
| DA1116 | *eat-2(ad1116) II* | - | CGC |
| VC222 | *raga-1(ok386) II* | *fln-2 (ot611)* in background | CGC |
| RB1206 | *rsks-1(ok1255) III* | - | CGC |
| MQ130 | *clk-1(qm30) III* | - | CGC |
| MQ887 | *isp-1(qm150) IV* | - | CGC |
| MQ1333 | *nuo-6(qm200) I* | - | CGC |
| TJ1052 | *age-1(hx546) II* | - | CGC |
| CF1041 | *daf-2(e1370) III* | - | Kenyon lab via the Dillin lab |
| TJ356 | *daf-16p::daf-16a/b::GFP* + *rol-6(su1006)* | DAF-16 overexpressor | CGC |
| WBM499 | *raga-1(ok386) II* | 6X outcrossed,*fln-2 (ot611)* in background | Mair Lab |
| SJ4100 | *zcIs13 [hsp-6::GFP]* *V* | *hsp-6* transcriptional reporter | CGC |
| SJ4058 | *zcIs9 [hsp-60::GFP + lin-15(+)]* *V* | *hsp-60* transcriptional reporter | CGC |
| WBM392 | *wbmIs31[acs-2p::GFP + rol-6(su1006)]* | *acs-2* transcriptional reporter | Mair Lab |
| WBM941 | *isp-1(qm150) IV; wbmIs31[acs-2p::GFP + rol-6(su1006)* | MQ887 X WBM392 | Mair Lab |
| WBM942 | *isp-1(qm150) IV; zcIs9 V* | MQ887 X WBM4058 | Mair Lab |
| WBM948 | *isp-1(qm150) IV; zcIs13 V* | MQ887 X WBM4100 | Mair Lab |
| WBM1186 | *repo-1(wbm28) IV* | 3XFLAG::REPO-1 (CRISPR) | Mair Lab |
| WBM1118 | *sfa-1(wbm32) IV* | 3xFLAG::SFA-1 (CRISPR) | Mair Lab |
| WBM960 | *repo-1(wbm14) IV* | REPO-1::GFP (CRISPR) | Mair Lab |
| WBM1101 | *sfa-1(wbm31) IV* | wrmScarlet::SFA-1 (CRISPR) | Mair Lab |
| WBM1117 | *repo-1(wbm14) sfa-1(wbm31) IV* | WBM960 X WBM1101 | Mair Lab |
| KH2235 | *lin-15(n765)ybIs2167[eft-3p::ret-1E4E5(+1)E6-GGS6-mCherry+ eft-3p::ret-1E4E5(+1)E6(+2)GGS6-GFP+lin-15(+)+pRG5271Neo] X* | *ret-1* splicing reporter worm | Gift from H. Kuroyanagi |

During revisions of the study, the authors were made aware of a secondary mutation in the background of the *raga-1(ok386)* mutant strain VC222. We subsequently identified it also in the background of the strain used in this study, WBM499. The secondary mutation is an allele of*fln-2*, a mutation found floating in the background of a subset of *C. elegans* mutant stocks obtained from the CGC that can have cofounding effects on *C. elegans* lifespan (*1*). We have confirmed that N2 (wildtype), DA1116 (*eat-2)*, and TJ1052 (*age-1*) strains are free of this secondary mutation, and have an abundance of evidence that this finding does not affect any of the conclusions presented. We have shown using independently generated alleles that confer loss of RAGA-1 function through targeted protein degradation (alleles that do not carry the secondary mutation) that *sfa-1* RNAi fully suppresses the longevity effects resulting from loss of RAGA-1 in the nervous system (*2*). And, importantly, we show here in this manuscript (Fig 4I, 4J) that both *sfa-1* RNAi and *repo-1* RNAi fully suppress the longevity effects resulting from knockdown of *raga-1* in wildtype N2 animals by *raga-1* RNAi when applied both early and later in life, but not later in life alone. These findings confirm that loss of RAGA-1 function in a wildtype background increases lifespan, and that the lifespan phenotype is fully suppressed by loss of splicing factor activity early in life.

**Lifespans**

All lifespans were conducted at 20°C. Lifespans were performed as described in (*3*). Graphpad Prism 9 was used to plot survival curves and calculate median lifespans. Lifespans were started with n=100 or 120 worms for each condition unless otherwise specified. Survival curves were compared, and p-values were calculated using the log-rank (Mantel-Cox) analysis method. Complete lifespan data is in Supplementary Table S11.

**RNA interference**

RNAi construct for *sfa-1*was obtained from the Ahringer RNAi and sequence verified (*4*). The *repo-1* RNAi was made by cloning the first 403 bases of the *repo-1* gene into the L4440 vector. RNAi experiments were done using *E.coli* HT115 bacteria on standard NGM plates containing 100µg/ml Carbenicillin. HT115 bacteria expressing RNAi constructs were grown overnight in LB supplemented with 12.5µg/ml Tetracycline and 100µg/ml Carbenicillin. The plates were seeded with 100 µl of the bacterial culture 48 hours before use. Respective dsRNA expressing HT115 bacteria were induced by adding 100µl IPTG (100mM) 1-2 hours before introducing worms to the plate. RNAi was induced from hatch or at Day 1 of adulthood as specified. Worms grown on empty vector HT115 RNAi bacteria are represented as control.

The sequences of the different RNAi constructs are listed below:

| *repo-1_FL_* RNAi | NNNNNNNNNNNNNNNNNTAGGGCGATTGGGTACCTCAATAGTACGGCTGATCATCGAGTCGTTCCAATTTGAATGCGACTTGTAAGAAGAATTGCTTCGTGTCTTTGTTCCACATCGTCCAAAATTTTTCAGATTTGTCAACTTCTCTTGATGGAATTTTGAATCCAATCGTTTCATACGGCTCAGCAGCAAACAAGAGGTATTGCCATCTCTTGTCTGGAGGCTGAATCTTTTGCTCATAAGCAGACATAAATCGATGACGTGGCGCAATACCGTCAGCAATCTCCGGATAATCAATTTGGAAGAGAAGTGCTTGCTGGCCAGCTCCTGGATCACGTTCTTTTGTTACCTTGTATCCAGGACGTCCGATTTTCACAAACTTTTTAGTTTCAACTGCAGCTTTCTGTGGAGCTGGTAGAAATGGTTGTTCAGATTGTTCTTTAGCGGCACGCCGTGCAAGATTCGCTTGATGCTTCTTTCCTTGTGTATGTGCCAAATAAGATCCTTCATTGTGGTGAAGAGTAAGACACAGCTTGCATTCGTACGTTCCAATGTGATTTCGCATGAAATACGGATCCTTTTGAAGATCAATTGTCTCTAGAGCCAACTGGCGGAGCCGTTCCCGTCGATCAACACCAGCATCGGCGGCCGAAGCCACTCCTCCGCTTCCCGTTTTTCCTCCAGCTCTGTTCTGAAAGTCCATGCTAGCCATGGAACCGGTGGATCCACTAGTTCTAGAGCGGCCGCCACCGCGGTGGAGCTCGAATTCATCGATGATATCAGATCTGCCGGTCTCCCTATAGTGAGTCGTATTAATTTCGAANAAGCCAGGTTGCTTCCTCGCTCACTGACTCGCTGCGCTCGGTCGTTCGGCTGNGGCGAGCGGTATCAGCTCACTCAANGNNGGTAATANGGTTATCCNNNNAATCAGGGGANANGCNNNAANAACATGTGAGCAAANGCCAGCAANNNCAGNANNGTAAAAGGCCGCGNTGCTGGNNTTTTCCNNNGGCTCCGNCCCCCNGACGAGCNTNNNNNN |
| --- | --- |
| *repo-1_short_* RNAi (or *repo-1* RNAi) | NNNNNNNNNNNNNNNNNNNNGGGCGATTGGGTACCGGAAGAGAAGTGCTTGCTGGCCAGCTCCTGGATCACGTTCTTNNNNNNNCTTGNATCCAGGACGTCCGATTTTCACAAACTTTTTAGTTTCAACTGCAGCTTTCTGTGGAGCTGGTAGAAATGGTTGTTCAGATTGTTCTTTAGCGGCACGCCGTGCAAGATTCGCTTGATGCTTCTTTCCTTGTGTATGTGCCAAATAAGATCCTTCATTGTTGTGAAGAGTAAGACACAGCTTGCATTCGTACGTTCCAATGTGATTTCGCATGAAATACGGATCCTTTTGAAGATCAATTGTCTCTAGAGCCAACTGGCGGAGCCGTTCCCGTCGATCAACACCAGCATCGGCGGCCGAAGCCACTCCTCCGCTTCCCGTTTTTCCTCCAGCTCTGTTCTGAAAGTCCATGCTAGCCATGGAACCGGTGGATCCACTAGTTCTAGAGCGGCCGCCACCGCGGTGGAGCTCGAATTCATCGATGATATCAGATCTGCCGGTCTCCCTATAGTGAGTCGTATTAATTTCGATAAGCCAGGTTGCTTCCTCGCTCACTGACTCGCTGCGCTCGGTCGTTCGGCTGCGGCGAGCGGTATCAGCTCACTCAAAGGCGGTAATACGGTTATCCACAGAATCAGGGGATAACGCAGGAAAGAACATGTGAGCAAAAGGCCAGCAAAAGGCCAGGAACCGTAAAAAGGCCGCGTTGCTGGCGTTTTTCCATANGCTCCGCCCCCCTGACGAGCATCACAAAAATCGACGCTCAAGTCAGANGTGGCGAAACCCGACAGGACTATAAAGATACCAGGCGTTTCCCCCTGGAAGCTCCCTCGTGCGCTCTCCTGTTCCGACCCTGCCGCTTACCGGATACCTGTCCGCCTTTCTCCCTTCGGGAAGCGTGGCGNNTTTNTCNNANNNNNNCACNNNGNANGGTATCTCANTNCGGNGTAGGTCGTTCGCNNCAAGCTGGGNTGNGTGCACGAANCCCCCNTTCAGCCNNACNNCTGCGCCTTATCCGGTAACTATCGTNTTGNNNTCCNACCCNGNANNA |
| *rbm-34* RNAi | NNNNNNNNNNNNNNNNTANNGGCGATTGGGTACCCTATGCCATCAGACGTCCCTTCTTTGCAGCCTTCTTCTTTGCAATGGCTTTCTTCGCTGACTTCTTCAAGGCACGACGATCATTTTGCTCAGTTGTTCTTTCTTTTTTGGTGGAGAACTTGAACTTGTGCAATTTTCCAGTAATTTCATTCTGGTTCTTCTTTCCGTGAGATGTTCGCTTTTTAGCAGTTTGGATCTTCGTCAAATGTCCCTTCTTCATAACTTTTGTAATGCGCAAGTCGCGTTTTTCCATTTTGATAGTCTCCATGCTCAATGCAAGACTCACAGAAGAATCTTGTTTGAAATTGACAAACGCGAATCCTTTTCCCTTTCCAGTATCTTTATCTCTGACAATTCTGACAGCCTCGACAGGTCCGATTTGTGCAGAGAAAAAAGTAATGAGAGCATCTTCGGTAATCTCGAACGGCAAGTTTCCAACAAAAATGGCCATATCCTTTCCAAATTCTTTCTTCTTACTTCCAACTTTGTCAACACGAATCACATGATCGTCCAGCTTGGTTCCATTGTACTTCAGAGCTTTCTCCACTGATTCTTCTGCTCCGAATTTCACATAGAACGTGAGAGAACTCTGTTTGTCATTCAATTTTCCTGTCAAATGAGTGACTCGTTTCGTCAATTTCTCATTCGCAGGAAGCAGATTTCTCATACGGACAGAGGAAATAGTTCCAAAGTCGGAGAAAATTCGACGGACTGATTTCTCGTTCATTGTCAATGGCATATTTCCAACAAAAACAGTCAGAGCATTTTCAGCAGCTGAAGCACGTGCATTTGACTTTTGAAGAGTTCTATTCTCTCTTTGCTTCGTTCTGTCTCGCTTTCCTTCCTTATTTTCATCTTTTTTCTCTTCTCCTTCTGTTNCNNNGCAACTTCTTCAGCAGCAGCAGCAGCATCAGCTNCNTCTCTTCNTCANCTTTTTTTTGGATCATGCTTTCTTCNTNNNTTTTCAATNACTTTATCTNCCNNNNNANTTCCTNNNNACTGCTGCNNCTTCTGACN |
| *sfa-1* RNAi (Ahringer Library) | TAGGGCGATTGGGTACCGGGCCCCCCCTCGAGGTCGACGGTATCGATAAGCTTGATTTGGAACAGTTGAGACATGAGAAAATTCAAGCTTTATTGAAGATTAATCCGAATTTCAAACCGCCCGCTGACTATCGGTATATATATAGGAAAATTTGAATTTTTCCGGCAAAAATGCGAGTTTTTGGTGAAAAATCACAGATTTTCAGAGTTTTCGGCTGAAAATTTGACCTTTTTCGCTGCAAATGTGTGATTCGGCTGAAAAATTCATTTTCAGCAATATTTAGCTTGAAACTACCGATTTTTTTGTTGAAAATCATTTAAAATTTCCGGATTTTCCGTTAAAAGTTGGAAATTCTTCAAATTTTTGCCCTTTGAATTTCGGCTAAAATTTGACCGAAAACACTAGAATTTTAAGCTGAAAACTGGATAAATTCCAGAAATTTGAACAATTGAGGTTTTATGGGATTTTTACGGATTTTTGACAAAAAAAACGGTAGTTTTCAAGCTGAAAATTGCTGTGAATAAATTTTTAAAATCTGAAACTCCAAAAATCTGTGATTTTTCACCAAAAACTCGCATTTTTGCCTAAGAAATTCAAATTTTAAGCGTAAATTATAATTTTTGCAATAAAATATCTAAAATTTCAATTTTTTTCAGTGCTCCAAACATCCGTCTACACGACAAAGTCTGGATTCCCCAGGAACAATTTCCTGATCTCAATTTTGTCGGCCTACTCATTGGTCCACGTGGAAATACGCTGAAAAGCTTGGAAGCTGAAACTGGAGCCAAGATTATTATCAGAGGAAAAGGATCTATAAAAGAGGGAAAATTGACGAATCGACTCGGACCGATGCCTGGNGAAAATGAGCCATTACATGCATATGTAACTGGAACTGATATGAATGTTATCAAAAAAGCATGTGAGAANATTAAACAAGTGATTGCTGAAGCCACTGCTCTGCCGGANACATGAGCTCAGGAAGCTGCAACTGANANACTCGCACTGTTGAATGGAACTTTCCGACCGGANGATTTNGCAAA |

**Sorting of splicing reporter worms**

KH2235 splicing reporter worms were thawed from -80°C stocks for each independent biological replicate. Worm populations were grown on NGM plates seeded with *E. coli* OP50-1 and age synchronized using 5% sodium hypochlorite solution. Around 2500 *C. elegans* eggs were placed on NGM plates with OP50-1 bacteria following the bleach process. On the first four days of adulthood, worms were transferred daily to fresh plates to remove progeny. At day 6 of adulthood, worm populations were assigned by visual separation to respective youthful and aged splicing pattern groups using a UV dissecting microscope (Zeiss Discovery V8). From each group separately, 100 were randomly selected using white light to include in survival analysis. Lifespans were set up as previously described. In addition, 8 randomly selected animals from each group were imaged as a representative population of the group as described previously (*4*).

For RNA extractions, about 200 animals were washed off plates using M9+0.01% Tween, washed twice using M9 buffer and flash-frozen in 250µl Qiazol in liquid nitrogen. For each biological replicate, duplicate samples were frozen. Samples were stored at -80°C and RNA extractions were performed in parallel for all biological replicates.

RNA extractions were performed using Qiagen miRNeasy micro kit and eluted in ddH_2_O.

Six independent biological replicates were sent for library preparation and RNA sequencing.

Three independent biological replicates were collected after the RNA sequencing following the same protocols for validation by qRT-PCR.

**RNA isolation and cDNA synthesis**

Total RNA was extracted using Qiazol (QIAGEN), column purified by RNeasy mini kit (QIAGEN) according to manufacturer’s instructions. cDNA was synthesized using SuperScript VILO Master mix (Invitrogen).

**Quantitative RT-PCR**

StepOne Plus instrument from Applied Biosystems was used to perform real-time qPCR experiments following the manufacturer’s instructions. The following Taqman assays from Life Technologies were used: *repo-1* (Ce02465496_g1), *rbm-34* (Ce02465498_g1), *acs-2* (Ce02486192_g1), *sod-3* (Ce02404518_gH),  *fat-3* (Ce02458252_g1), *cpt-5* (Ce02419317_g1) , *fat-7*(Ce02477067_g1), *acdh-1*(Ce02408341_g1), *acdh-2*(Ce02432818_g1), *gst-10*(Ce02504848_m1) . For each qPCR reaction, ~5ng of cDNA was used. Relative expression differences were calculated with the comparative 2ΔΔCt method using Y45F10D.4 (Ce02467253_g1) as the endogenous control. For each gene in each strain, fold-change relative to the average of wild type control group was calculated and statistical significance evaluated using Welch’s t test. Graphpad Prism 9 was used for all statistical analysis and graph plotting.

**Semi-quantitative RT-PCR of *tos-1* to visualize alternative splicing event**

*tos-1* was amplified using PCR conditions and primers as described in (*4*). Apex Taq RED (Genesee Scientific) was used for amplification. 1 Kb Plus DNA ladder (Invitrogen) was used as molecular weight reference. Following PCR, samples were resolved on a 2% EtBr-stained agarose gel. Gels were imaged on ChemiDoc MP (BioRad).

**Microinjection and CRISPR/Cas9 mediated gene editing**

All CRISPR edits were performed using the CRISPR protocol developed by (*5*). Briefly, homology repair templates were amplified by PCR using primers that introduced a minimum stretch of 35 bp homology arms flanking the site of insertion at both ends. The CRISPR injection mix contained 2.5 μl tracrRNA (4 μg/μl), 0.6 μl *dpy-10*crRNA (2.6 μg/μl), 0.5 µl target gene crRNA (2.6 μg/μl), 0.25 μl *dpy‐10*ssODN (500 ng/μl), homology repair template (200 ng/μl final in the mix), 0.375 μl Hepes pH 7.4 (200mM), 0.25 μl KCl (1M) and RNase free water to make up the volume to 8 µl. 2 μl purified Cas9 (12 μg/μl) was added at the end, mixed by pipetting, spun for 2 min at 13000 rpm and incubated at 37^o^C for 10 minutes. *dpy-5* was used as a co-injection marker instead of *dpy-10* in case the edits were made on Chromosome II. Mixes were microinjected into the germ line of day 1 adult hermaphrodites using standard protocol. Screening worms and genotyping was performed as described earlier (*6*). Worms generated using CRISPR were outcrossed at least six times before being used for experiments to remove the co–injection marker phenotype and other off-target edits.

**Microscopy**

For DIC and fluorescence imaging of REPO-1::GFP and wrmScarlet::SFA-1 at different stages, worms were anesthetized in 0.1 mg/ml tetramisole in 1X M9 buffer on empty NGM plates and mounted on 2% agarose pads on glass slides. They were imaged using the Apotome.2-equipped Imager M2 microscope with an Axiocam camera. For imaging of hsp-6 and hsp-60, on NGM plates without bacteria until no movement was detectable, aligned to groups accordingly and subsequently imaged on a Zeiss Discovery V8 microscope equipped with an Axiocam camera. Exposure times were kept constant for all imaging experiments involving the splicing reporter.

**Western Blotting and Quantification**

3XFLAG::*repo-1* worms were egg laid on *E. coli* HT115 bacteria to get a synchronized population of worms. To age them up until Day 15, worms were transferred on days 1, 2, 3, 5 and 8 to separate them from their progeny. For Day 1, Day 5, Day 10 and Day 15 samples, approximately 200 adults per sample were collected in triplicates in M9 buffer and snap frozen in liquid nitrogen. For larval stages, approximately 40 animals were made to egg lay over a 12-14 hour period on each plate. 10 such plates were pooled after 24 or 48 hours to get one replicate of L1-L2 or L3-L4 stage sample respectively. To make worm lysates, RIPA buffer with protease inhibitors (Sigma #8340) were added and samples were lysed via sonication at Amplitude 60 10sec ON / 10sec OFF for a total of 3 times (Qsonica Q700) and this complete cycle was repeated 3 times. SDS-PAGE was performed by running 20 µg of protein per lane on a 4-12% TrisGlycine gradient gel (Thermo Fisher Scientific, #XP04122BOX). Proteins were transferred to Nitrocellulose membranes (BioRad # 162-0112) and blocked with 5% BSA in TBST. They were stained with Ponceau-S Stain, 0.1% Solution (G Biosciences #89167-800) and imaged. Ponceau was washed off with 1XTBST and blots were incubated with primary antibody ANTI-FLAG M2 mouse monoclonal (Sigma Aldrich #F1804, 1:2000). They were then washed in 1XTBST and probed with secondary anti-HRP linked mouse antibody (Cell Signaling #7076, 1:5000). Blots were developed using ECL substrate (GE Healthcare # 95038-560).  Bands were visualized using a Gel Doc system (Bio Rad) and Image Lab software (Version 4.1). Blots were stripped using Stripping Buffer (Thermofisher # PI46430) according to manufacturer’s guidelines and re-probed with beta actin (Abcam, #8226, 1:1000). Quantification of the bands was done using ImageJ (Version 1.52a) and plotted using Graphpad Prism 9.

**RNA-Sequencing**

Libraries were prepared using Roche Kapa stranded mRNA HyperPrep sample preparation kits from 100ng of purified total RNA according to the manufacturer’s protocol. The finished dsDNA libraries were quantified by Qubit fluorometer, Agilent TapeStation 2200, and RT-qPCR using the Kapa Biosystems library quantification kit according to manufacturer’s protocols. Uniquely indexed libraries were pooled at an equimolar ratio and sequenced on an Illumina NextSeq500 with paired-end 75bp reads by the Dana-Farber Cancer Institute Molecular Biology Core Facilities.

**Differential Gene Expression Analysis**

All samples were processed using an RNA-seq pipeline implemented in the bcbio-nextgen project (<https://bcbio-nextgen.readthedocs.org/en/latest/>). Raw reads were examined for quality issues using FastQC (v0.11.8) (<http://www.bioinformatics.babraham.ac.uk/projects/fastqc/>) to ensure library generation and sequencing were suitable for further analysis.

To perform additional quality checks of the data, all reads were aligned to Wormbase assembly WBcel235, release WS272 of the C. elegans genome (Project PRJNA13578 (N2 strain)) using STAR (v. 2.6.1d) (*7*). Alignments were checked for evenness of coverage, rRNA content, genomic context of alignments (for example, alignments in known transcripts and introns), complexity and other quality checks using a combination of FastQC, Qualimap (*8*),

<http://doi.org/10.1093/bioinformatics/bts503>], MultiQC (<https://github.com/ewels/MultiQC>) and custom tools.

To quantitate the reads corresponding to each transcript, quasi alignment was performed using Salmon (v. 0.14.2) (*9*), outputting the transcripts Per Million (TPM) measurements per isoform. Differential expression at the gene level was called with DESeq2 (*10*), using counts per gene estimated from the Salmon quasi alignments by tximport. The differential effect of *repo-1* knockdown on the mutants *raga-1(ok386)*, *eat-2(ad1116)*, and *clk-1(qm30)* relative to *age-1(hx546)*was explored using the design formula: ~ repo1_effect + treatment + repo1 effect:treatment.

The DEGReport (*11*)[ <http://lpantano.github.io/DEGreport/>] package was used to identify gene clusters that change similarly upon knockdown of repo-1 using a hierarchical correlation clustering approach. UpSet plots of shared differentially expressed genes between analyses were generated using the UpSetR R package (*12*).  Lists of differentially expressed (DE) genes in LL vs SL worm RNA-Seq or groups of DE genes with similar expression changes with *repo-1* knockdown in REPO-1 RNA-Seq identified by DEGReport were examined for enrichment using  WormCat (*13*).

**Splicing and Differential Isoform Usage Analysis of SL vs LL worm sub-populations**

Differential transcript usage and local alternative splicing events were identified using SUPPA2 (*14*, *15*). The TPM values output from Salmon were used as input to SUPPA2, and differential splicing analysis was performed using the empirical method. The p-values were corrected for multiple testing and an alpha of 0.05 was used for identification of significant events. An event was not tested if none of the transcripts associated with the event were expressed for any sample or if one or more transcripts of the event was not quantified for any sample.

The genes corresponding to significant events were examined for over-representation analysis with clusterProfiler (*16*). The background set of genes represent all spliced genes that were tested for differential splicing of events (e.g. spliced genes that were expressed in all samples). The significant genes were de-duplicated for genes that corresponded to more than one significant event. Enriched processes represent those biological processes enriched for genes that are significantly differentially spliced compared to all, expressed spliced genes. The biological processes tested were defined by the gene sets obtained from Wormcat (*13*) and were tested separately at each hierarchical level.

***Genome and transcriptome reference files and annotations download links:***

**Genome:** <ftp://ftp.wormbase.org/pub/wormbase/releases/WS272/species/c_elegans/PRJNA13758/c_elegans.PRJNA13758.WS272.genomic.fa.gz>

**Transcriptome:** <ftp://ftp.wormbase.org/pub/wormbase/releases/WS272/species/c_elegans/PRJNA13758/c_elegans.PRJNA13758.WS272.canonical_geneset.gtf.gz>

**Annotations:** <ftp://ftp.wormbase.org/pub/wormbase/releases/WS272/species/c_elegans/PRJNA13758/annotation/>

**SRS microscopy imaging, *C. elegans* sample preparation and image quantification**

For the SRS microscope system, pulsed Pump (tunable from 790 to 990 nm) and Stokes (1045 nm) beams were provided by Insight X3 femtosecond laser (Spectra-Physics), and spatiotemporally overlapped by a Spectral Focus Timing and Recombination Unit (SF-TRU, Newport). The intensity of Stokes beam is modulated at 20 MHz by an electro-optic modulator (EOM, 4103, New Focus). Overlapping pump and Stokes beams were emitted from the port of SF-TRU and coupled into a multi photon laser scanning microscope (FVMPE-RS, Olympus). After passing through the sample, the forward going Pump and Stokes beams were collected by an air condenser. A flip mirror was used to direct the transmitted laser to a photodiode module, which includes a telescope to relay and change the beam size to fit the area of the photodiode, as well as an optical filter to remove the modulated Stokes beam and let the pump beam transmit for the detection of stimulated Raman loss signal. The output current from the photodiode was terminated, filtered, and demodulated by a lock-in amplifier (SRS Detection Module, APE) at 20 MHz to ensure shot noise-limited detection sensitivity. The lock-in amplifier output was then fed into the analog box of FVMPE-RS microscope system to process the SRS signal. For lipid imaging, CH_2_ signals were detected at 2845 cm^-1^. To achieve this, pump beam was set at 805 nm and the delay stage was positioned at 33.550 nm. For both pump and Stokes beams, 10% laser power was used. A 20x air objective (UPlanSAPO, 0.75 N.A., Olympus) was used for imaging. The microscope was controlled by Olympus Fluoview software. SRS microscopy images were quantified using ImageJ software (NIH). Polygon selection tool was used to select the area to be quantified and average pixel intensity was calculated with the “analyze-measure” command. After subtracting the background intensity, all measurements were averaged to obtain mean and standard deviation. In each imaging session, approximately 20-30 worms were immobilized with 1% sodium azide on 2% agarose pads on glass microscope slides and 10-20 worms, which have anterior intestine in focus, were imaged. Mutant lipid levels were normalized to those of wild-type worms and relative SRS signals are shown in box plots. Wild-type and mutant worms were bleached on plates with HT115 bacteria and their progeny were used for adult-egg laying on plates with HT115 bacteria transformed with either empty L4440 vector or vector carrying RNAi against *sfa-1* or *repo-1*. Embryos were grown at 20°C, on these plates, until larval L4 stage, when they are synchronized again and imaged 24 hours later. For *pod-2* RNAi and adult-onset *repo-1* RNAi wild-type and mutant worms were bleached on plates with HT115 bacteria and their progeny were used for adult-egg laying on plates with HT115 bacteria transformed with empty L4440, allowed to grow until Day 1 of adulthood then transferred to either empty L4440 vector or vector carrying RNAi against *pod-2* or *repo-1*, transferred daily to fresh plates to avoid progeny and imaged as described on Day 4 of adulthood.

**Lipid extraction**

About 1000 worms for each condition were washed off plates using M9+0.01% Tween, washed twice using M9 buffer and flash-frozen in ~200 μl M9 in liquid nitrogen. Samples were stored at -80°C. 4 biological replicates were collected, and lipid extraction was done in parallel among all replicates. Lipids were extracted according to Folch’s method. Briefly, nematodes were suspended in 500 µL water and Lyzed by snap-freezing (in liquid nitrogen)/thawing (using an ultrasound water bath for 5 min) repeatedly, and finally extracted with chloroform: methanol (2:1). The lower organic phase was transferred to fresh 4 mL glass vials and the organic phase was re-extracted with additional 4ml chloroform. After centrifugation, the organic phases were combined and evaporated. The residue was reconstituted in 150ul chloroform: methanol (2:1, v/v) and at − 80 °C until analysis. Internal standard SPLAH mix spiked in prior to extraction and was used for normalization. Furthermore, the organic phase of each sample was normalized by total soluble protein amounts and measured by BCA assay (Thermo Scientific, 23225, Waltham, MA).

**Liquid-chromatographic mass spectrometric conditions**

UltiMate 3000 with the standard binary system (ThermoFisher Scientific) and an autosampler were used for this study. The separation was performed using CSH C18 (100 × 2.1 mm, 1.7 μm, Waters) columns. The column oven temperature suitable for all columns was 50 °C. The multi-step gradient elution was carried out using different ratios of eluents A (ACN: water, 60:40, v/v) and B (IPA: ACN, 90:10, v/v), both containing 10 mM ammonium formate and 0.1% formic acid. Separation was achieved using the following gradient: 0 −3 min isocratic elution with 30% B, 3–5 min, 30–55% B; 5.1–14 min, 55–65% B; 14.1–21 min, 65–99% B; 21–24 min, 99% B; 24–28 min, 99–30%; 28–30 min, 30% B for column washing and equilibration. The flow rate was set to 350 μL/min, the temperature of the sample tray was set to 4 °C and the injection volume was 5 μL.

For the untargeted lipidomic analysis, a Q Exactive hybrid quadrupole-Orbitrap mass spectrometer (ThermoFisher Scientific, USA) was employed. The experiments were performed in positive and negative ion modes using a HESI source. The flow rates of sheath gas and sweep gas for both polarities were adjusted to 50 and 1 (arbitrary units), while the auxiliary gas rate was 15 for positive and 15 for negative. For both ionization modes, the spray voltage, the capillary temperature, and the heater temperature were maintained at 3 kV, 300 °C, and 320 °C, respectively. The S-Lens RF level was set at 50. The Orbitrap mass analyzer was operated at a resolving power of 140,000 in full-scan mode (scan range: 133.4–2000 *m/z*; automatic gain control target: 1e^6^) and of 35,000 in the Top10 data-dependent MS^2^ mode (HCD fragmentation with stepped normalized collision energy: 25 and 30 in positive ion mode, and 25, 30 and 35 in negative ion mode; Injection time: 100 ms; Isolation window: 1*m/z*; automatic gain control target: 1e^5^) with dynamic exclusion setting of 15.0 s.

*Data processing*

All MS data were acquired and processed using the software package Xcalibur 3.0. LipidSearch software version 4.1 (Mitsui Knowledge Industry, Tokyo, Japan) was used for lipid molecular species identification and quantification. Data processing parameters were target database: QExactive; precursor tolerance: 5 ppm; product tolerance: 20 ppm; product ion threshold: 5%; m-score threshold: 1; Quan *m/z* tolerance: ±5 ppm; Quan RT (retention time) range: ±0.25 min; use of main isomer filter and ID quality filters A, B; Adduct ions: +H and +NH_4_ for positive ion mode, and −H, +HCOO and −2H for negative ion mode. The lipid classes selected for the search were: LPC (lysophosphatidylcholine), PC (phosphatidylcholine), LysoPE (lysophosphatidylethanolamine), PE (phosphatidylethanolamine), LysoPS (lysophosphatidylserine), PS (phosphatidylserine), LysoPG (lysophosphatidylglycerol), PG (phosphatidylglycerol), LysoPI (lysophosphatidylinositol), PI (phosphatidylinositol), LysoPA (lysophosphatidic acid), PA (phosphatidic acid), SM (sphingomyelin), MG (monoacylglycerol), DG (diacylglycerol), TG (triacylglycerol), CL (cardiolipin), So (sphingosine), Cer (ceramides), Che (cholesterol ester). The same lipid annotations within ±0.1 min were merged into the aligned results. Finally, only lipid species that have less than 25 CoV% (coefficient of variation) in the QC (quality control) were considered for further analysis.

**Enhanced Crosslinking Immunoprecipitation (eCLIP) in MEFs and Worms**

eCLIP was performed by Eclipse Bioinnovations Inc (San Diego, www.eclipsebio.com) according to the published eCLIP protocol (*17*). For each replicate (N=2), 20 million MEFs were UV crosslinked (UVC-515 Ultralum) at 400 mJoules/cm2 with 254-nm radiation, and snap frozen in liquid nitrogen. Cells were lysed and treated with RNase I to fragment RNA as previously described. SF1 (Bethyl: Cat No. A303-213A, Lot No. A303-213A-1) and SF3A2 (Bethyl: Cat No. A304-821A, Lot No. A304-821A-1) antibodies were used for immunoprecipitation of the proteins respectively. Only the region from 65 kDa to 140 kDa was excised for eCLIP. RNA adapter ligation, immunoprecipitation-western blotting, reverse transcription, DNA adapter ligation and PCR amplification were performed as previously described.

For *C. elegans* eCLIP (N=1), 50,000 worms or 50uL packed worms (~1mg of total protein) was grown. Worms were obtained from an egg lay of CRISPR tagged 3XFLAG::SFA-1 (WBM1118) and 3XFLAG::REPO-1 (WBM1186) on 10 cm plates seeded with HT115 bacteria. At Day 1 of adulthood, they were transferred to a 15 ml centrifuge tube in M9+0.01% Tween and washed gently 3 times with 10mL M9 at room temperature. They were resuspended in 5 ml fresh M9 and transferred to NGM plates without bacteria. After the worms dispersed uniformly, the plates were placed on leveled ice, plate lid removed followed by crosslinking (UVC-515 Ultralum) at 254-nm UV with an energy setting of 500 mJoules/cm2. Immobilized worms were transferred to a 2mL round bottom microcentrifuge tube using M9 and spun for 30 seconds at 3,000xg. Excess M9 was aspirated out and the worm pellet flash frozen in liquid nitrogen. Monoclonal Anti-FLAG M2 antibody (Sigma: Cat No. F1804-1MG, Lot No. SLBX2256) was used for immunoprecipitation of FLAG::SFA-1 and FLAG::REPO-1 and eCLIP was performed by excising the 80-155kDa region for SFA-1 and 32-115kDa for REPO-1.

**REFERENCES FOR SUPPLEMENTARY MATERIAL**

1. Y. Zhao, H. Wang, R. J. Poole, D. Gems, A fln-2 mutation affects lethal pathology and lifespan in C. elegans. *Nat. Commun.* **10**, 5087 (2019).

2. H. J. Smith, A. Lanjuin, A. Sharma, A. Prabhakar, E. Nowak, P. G. Stine, R. Sehgal, K. Stojanovski, B. D. Towbin, W. B. Mair, Neuronal mTORC1 inhibition promotes longevity without suppressing anabolic growth and reproduction in C. elegans. *PLoS Genet.* **19**, e1010938 (2023).

3. K. Burkewitz, I. Morantte, H. J. M. Weir, R. Yeo, Y. Zhang, F. K. Huynh, O. R. Ilkayeva, M. D. Hirschey, A. R. Grant, W. B. Mair, Neuronal CRTC-1 governs systemic mitochondrial metabolism and lifespan via a catecholamine signal. *Cell* **160**, 842–855 (2015).

4. C. Heintz, T. K. Doktor, A. Lanjuin, C. C. Escoubas, Y. Zhang, H. J. Weir, S. Dutta, C. G. Silva-García, G. H. Bruun, I. Morantte, G. Hoxhaj, B. D. Manning, B. S. Andresen, W. B. Mair, Corrigendum: Splicing factor 1 modulates dietary restriction and TORC1 pathway longevity in C. elegans. *Nature* **547**, 476 (2017).

5. A. Paix, A. Folkmann, D. Rasoloson, G. Seydoux, High Efficiency, Homology-Directed Genome Editing in Caenorhabditis elegans Using CRISPR-Cas9 Ribonucleoprotein Complexes. *Genetics* **201**, 47–54 (2015).

6. C. G. Silva-García, A. Lanjuin, C. Heintz, S. Dutta, N. M. Clark, W. B. Mair, Single-Copy Knock-In Loci for Defined Gene Expression in Caenorhabditis elegans. *G3*  **9**, 2195–2198 (2019).

7. A. Dobin, C. A. Davis, F. Schlesinger, J. Drenkow, C. Zaleski, S. Jha, P. Batut, M. Chaisson, T. R. Gingeras, STAR: ultrafast universal RNA-seq aligner. *Bioinformatics* **29**, 15–21 (2013).

8. F. García-Alcalde, K. Okonechnikov, J. Carbonell, L. M. Cruz, S. Götz, S. Tarazona, J. Dopazo, T. F. Meyer, A. Conesa, Qualimap: evaluating next-generation sequencing alignment data. *Bioinformatics* **28**, 2678–2679 (2012).

9. R. Patro, G. Duggal, M. I. Love, R. A. Irizarry, C. Kingsford, Salmon provides fast and bias-aware quantification of transcript expression. *Nat. Methods* **14**, 417–419 (2017).

10. M. I. Love, W. Huber, S. Anders, Moderated estimation of fold change and dispersion for RNA-seq data with DESeq2. *Genome Biol.* **15**, 550 (2014).

11. L. Pantano, DEGreport: Report of DEG analysis. *New Jersey, NJ: R package version* **1** (2019).

12. J. R. Conway, A. Lex, N. Gehlenborg, UpSetR: an R package for the visualization of intersecting sets and their properties. *Bioinformatics* **33**, 2938–2940 (2017).

13. A. D. Holdorf, D. P. Higgins, A. C. Hart, P. R. Boag, G. J. Pazour, A. J. M. Walhout, A. K. Walker, WormCat: An Online Tool for Annotation and Visualization of Caenorhabditis elegans Genome-Scale Data. *Genetics* **214**, 279–294 (2020).

14. J. L. Trincado, J. C. Entizne, G. Hysenaj, B. Singh, M. Skalic, D. J. Elliott, E. Eyras, SUPPA2: fast, accurate, and uncertainty-aware differential splicing analysis across multiple conditions. [Preprint] (2018). https://doi.org/10.1186/s13059-018-1417-1.

15. G. P. Alamancos, A. Pagès, J. L. Trincado, N. Bellora, E. Eyras, Leveraging transcript quantification for fast computation of alternative splicing profiles. *RNA* **21**, 1521–1531 (2015).

16. G. Yu, L.-G. Wang, Y. Han, Q.-Y. He, clusterProfiler: an R Package for Comparing Biological Themes Among Gene Clusters. *OMICS* **16**, 284–287 (2012).

17. E. L. Van Nostrand, G. A. Pratt, A. A. Shishkin, C. Gelboin-Burkhart, M. Y. Fang, B. Sundararaman, S. M. Blue, T. B. Nguyen, C. Surka, K. Elkins, R. Stanton, F. Rigo, M. Guttman, G. W. Yeo, Robust transcriptome-wide discovery of RNA-binding protein binding sites with enhanced CLIP (eCLIP). *Nat. Methods* **13**, 508–514 (2016).
